# Construction of PANoptosis‐Inhibiting Carbonized Polymer Dots via Machine Learning Potential for Mitigating Chemodrug‐Induced Nephrotoxicity

**DOI:** 10.1002/advs.202512196

**Published:** 2025-12-12

**Authors:** Xinchen Liu, Jiaxin Zhang, Xiangyu Yan, Nuo Li, Yu‐Chao Dong, Zihao Wang, Daowei Li, Yong Du, Huan Wang

**Affiliations:** ^1^ Jilin Provincial Key Laboratory of Tooth Development and Bone Remodeling School and Hospital of Stomatology Jilin University Changchun 130021 P. R. China; ^2^ State Key Laboratory of Rare Earth Resource Utilization and Laboratory of Chemical Biology Changchun Institute of Applied Chemistry Chinese Academy of Sciences Changchun 130022 P. R. China; ^3^ State Key Laboratory of Powder Metallurgy Central South University Changsha 410083 P. R. China; ^4^ National Engineering Research Center for the Emergency Drug Beijing 100039 P. R. China; ^5^ Orthopedic Institute of Jilin Province Orthopedic Medical Center The Second Hospital of Jilin University Changchun 130041 P. R. China; ^6^ Department of Cariology and Endodontics Nanjing Stomatological Hospital Medical School of Nanjing University Nanjing 210008 P. R. China

**Keywords:** bioactive carbonized polymer dots, chemodrug‐induced nephrotoxicity, computer‐assisted design, metadynamics, PANoptosis inhibition

## Abstract

Chemotherapy‐induced nephrotoxicity, particularly acute kidney injury (AKI), is a severe complication that is driven by multiple regulated cell death (RCD) pathways. However, current nephroprotective strategies predominantly focus on apoptosis inhibition by relieving oxidative stress. PANoptosis is a newly discovered form of RCD pathway incorporating key features of pyroptosis, apoptosis, and necroptosis, providing a potential target for synergistic multi‐mechanistic nephroprotection. Herein, the enhanced sampling method is introduced for molecular dynamics based on trained machine learning force fields to guide the construction of bioactive carbonized polymer dots (Lu‐CDs) with specific pharmacophoric moieties of the flavonoid compound. Unlike conventional carbonized polymer dots with solely antioxidative activity, Lu‐CDs demonstrate both radical scavenging and PANoptosis‐inhibiting activities. In chemodrug‐induced AKI mice, a very low dose of Lu‐CDs can elicit prolonged renal accumulation and superior nephroprotective efficacy compared to the small‐molecule flavonoid compound and the antioxidative carbonized polymer dots. The findings of this study highlight a design strategy for bioactive nanodrugs based on the machine learning force fields for molecular dynamics and propose PANoptosis inhibition as a promising approach to mitigate chemodrug‐induced nephrotoxicity.

## Graphical abstract

An enhanced sampling method is developed for molecular dynamics based on trained machine learning force fields to guide the construction of PANoptosis‐inhibiting carbonized polymer dots (Lu‐CDs) derived from a flavonoid compound for treating chemodrug‐induced nephrotoxicity. These Lu‐CDs elicit superior nephroprotective efficacy compared to their flavonoid precursor, validating the bioactive nanodrug design strategy.

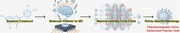

## 1 Introduction

Chemotherapy is a widely utilized and effective therapeutic modality for cancer treatment, yet adverse effects on normal tissues often constrain its efficacy.^[^ ^1^, ^2^, ^3^ ^]^ Among these adverse effects, chemotherapy‐induced nephrotoxicity, particularly acute kidney injury (AKI), represents a frequent and lethal complication that severely affects patients undergoing chemotherapy.^[^ ^4^, ^5^, ^6^ ^]^ Many cytotoxic chemodrugs are renally excreted and can induce structural and functional damage to renal cells.^[^ ^7^ ^]^ Meanwhile, the impaired renal function can, in turn, exacerbate the accumulation of cytotoxic chemodrugs, thus further aggravating kidney injury and forming a vicious circle. However, current clinical settings lack specific interventions for effective AKI management, with strategies primarily limited to supportive treatments, including hydration as well as renal replacement therapy.^[^ ^8^ ^]^ Recently, antioxidant‐based therapeutic approaches have emerged as a predominant strategy for AKI management because the excessive reactive oxygen species (ROS) production has been regarded as a major mediator of renal dysfunction through the induction of apoptotic cell death. Since then, numerous kidney‐specific antioxidative agents, such as graphene‐based materials, DNA nanostructures, supramolecular structures, and metal oxide nanoclusters, have been developed to eliminate the overproduced ROS and protect against apoptosis in the kidneys for mitigating chemodrug‐induced nephrotoxicity, yet the efficacy of treatment is still far from satisfactory.^[^ ^8^, ^9^, ^10^, ^11^, ^12^ ^]^

In addition to apoptotic cell death, emerging evidence indicates that other vital regulated cell death (RCD) forms are also pertinent to the pathogenesis of kidney diseases, including chemodrug‐induced AKI.^[^ ^13^ ^]^ Consequently, an optimal therapeutic strategy should simultaneously target multiple RCD pathways to achieve comprehensive nephroprotection. PANoptosis has recently been identified as a newly discovered form of RCD incorporating key features of pyroptotic, apoptotic, and necroptotic pathways.^[^ ^14^, ^15^, ^16^, ^17^, ^18^, ^19^, ^20^, ^21^, ^22^, ^23^ ^]^ Distinct from other single forms of cell death pathways, PANoptosis demonstrates a highly interconnected signaling network that simultaneously activates the above three pathways, realizing synergistic multi‐mechanistic cell death. In this regard, PANoptosis inhibition in renal cells represents a potential therapeutic target for ameliorating chemodrug‐induced nephrotoxicity. While investigations into PANoptosis‐related organ injury therapies remain in their infancy, current studies have predominantly focused on elucidating the PANoptosis‐regulating mechanisms of bioactive small molecules.^[^ ^24^ ^]^ Notably, there remains a conspicuous lack of kidney‐specific PANoptosis inhibitors to effectively mitigate AKI.

Peroxisome proliferator‐activated receptor alpha (PPARα) is a critical nuclear receptor that is highly expressed in organs such as the liver, heart, kidneys, and skeletal muscles.^[^ ^25^, ^26^ ^]^ It regulates the transcription of various genes involved in inflammation, oxidative stress, lipid metabolism, and energy homeostasis. Previous studies have demonstrated that the expression of PPARα is significantly reduced following AKI, and restoring PPARα levels effectively confers protective effects against AKI.^[^ ^27^, ^28^ ^]^ In the kidneys, the activation of the NLRP3 inflammasome serves as a central mechanism in renal pathophysiology. PPARα can modulate NLRP3 inflammasome activity, thereby mitigating inflammation.^[^ ^29^ ^]^ Flavonoids exhibit antioxidative and anti‐inflammatory properties, offering protective effects against nephrotoxicity, apoptosis, fibrosis, and inflammation‐related renal dysfunction. Among them, luteolin stands out for its potent antioxidative and anti‐inflammatory activities,^[^ ^30^ ^]^ which can be attributed to its intrinsic antioxidative structure and regulatory role in NLRP3‐mediated apoptosis and pyroptosis.^[^ ^31^, ^32^ ^]^ Recent studies have revealed that NLRP3 not only participates in inflammasome assembly but also collaborates with caspase‐8 in the formation of the more intricate and functionally significant PANoptosome complex, contributing to PANoptosis.^[^ ^16^ ^]^ Hence, optimizing the renal specificity of luteolin and elucidating its biological mechanisms will potentiate the therapeutic effects of chemodrug‐induced nephrotoxicity.

Recently, carbonized polymer dots (CPDs) have emerged as promising candidates in biomedicine due to their widespread sources, high biosafety, and customizable bioactive properties.^[^ ^33^, ^34^, ^35^, ^36^, ^37^, ^38^, ^39^, ^40^, ^41^, ^42^, ^43^ ^]^ Remarkably, the preserved structural moieties and functional groups on CPDs confer desirable precursor‐dependent functionalities.^[^ ^44^, ^45^, ^46^, ^47^, ^48^, ^49^ ^]^ Furthermore, adequately engineered ultrasmall CPDs exhibit ideal biodistributions, characterized by prolonged body retention and tissue‐targeting specificity.^[^ ^8^, ^50^, ^51^, ^52^, ^53^ ^]^ Consequently, we envisaged that the transformation of PANoptosis‐inhibiting compounds into CPDs with inherited pharmacophoric moieties and favorable biodistributions could effectively alleviate chemodrug‐induced nephrotoxicity through blocking the synergistic mechanisms of multiple cell death pathways.

The advent of machine learning in force field development has led to an increase in the number of applications for studying molecular dynamics (MD) at larger and longer timescales, which has been employed to guide material design and synthesis.^[^ ^54^, ^55^, ^56^ ^]^ In the present study, we introduced the enhanced sampling method for MD based on trained machine learning force fields to instruct the synthesis of CPDs (Lu‐CDs) with required pharmacophoric moieties by using the bioactive flavonoid compound luteolin as the starting precursor. Lu‐CDs elicit high PANoptosis‐inhibiting activity and antioxidative activity because of the enriched pharmacophoric moieties on Lu‐CDs, whereas the conventional CPDs, composed of only graphitic carbon cores and oxygenated groups, show solely antioxidative activity with no obvious PANoptosis‐inhibiting effects. In the mouse model of cisplatin‐induced AKI, Lu‐CDs could effectively accumulate in the kidneys over 3 days and inhibit PANoptosis, exerting superior therapeutic effects than those of the conventional CPDs and the precursor luteolin. This study not only develops an enhanced sampling method for MD based on trained machine learning force fields to guide the synthesis of bioactive nanodrugs but also proposes a new therapeutic strategy for effectively counteracting chemotherapy‐induced nephrotoxicity by targeted PANoptosis inhibition.

## 2 Results and Discussion

**Figure 1:**
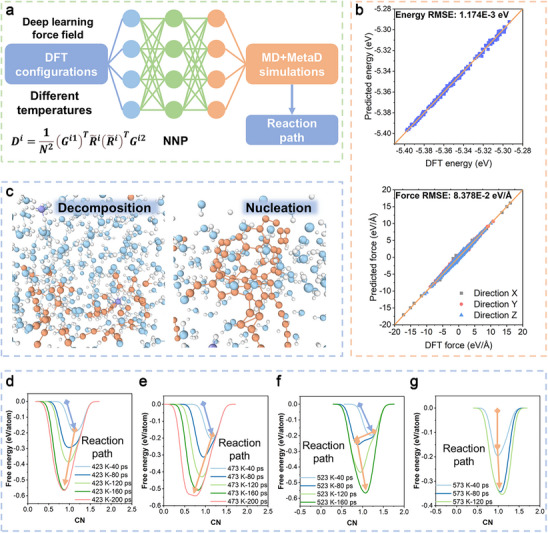
ML‐FF‐based MD simulations for the hydrothermal reaction design. a) The schematic illustration of the present simulating process. The collected DFT configurations are applied in deep learning training to obtain FF. MD and MetaD are then used to simulate the present hydrothermal reaction. b) The fitness of the ML results compared with the DFT results of energies and forces. c) The snapshots of decomposition and nucleation during the present simulation. Light blue, orange, white, and light violet represent the O, C, H, and N, respectively. d‐g) The calculated free energy surfaces based on the present MetaD calculations, and the time interval between different lines is 40 ps. The Cornflower Blue arrows represent agglomeration, while the Sandy Brown arrows mean decomposition.

To effectively transform luteolin into bioactive CPDs composed of a carbon core and conjugated bioactive moieties of luteolin to elicit desired functionalities, a hydrothermal reaction was designed by molecular dynamics (MD) simulations at the outset. The force fields (FF) in this large system were generated from machine learning methods based on DeepMD software according to configurations originating from the density functional theory (DFT) methods.^[^ ^57^ ^]^ The details of the settings are in the method section, and all the reaction processes are presented in **Figures** **1** and S1 (Supporting Information). Machine learning for generating force fields is an efficient method for simulating large systems, which is not supported by the first‐principles method. In the present design, we collected the configurations containing energies and forces originating from Ab‐initio Molecular Dynamics (AIMD) to train the FF. Figure 1b is the linear fitness of energies and forces between the DFT results and the machine learning (ML) predicted results, in which the root mean square error (RMSE) was 1.174 × 10^−3^ eV and 8.378 × 10^−2^ eV Å^−1^ of energies and forces. The outcomes of this study indicated that the machine learning force fields (ML‐FF) were credible for the following MD simulations. Then, the simulated snapshots of the hydrothermal reactions are presented in Figure S1 (Supporting Information) under various temperatures at different time points.

To achieve more effective simulations, the metadynamics (MetaD) method was employed, and the coordination numbers (CN) were identified as the collective variables (CV) for enhanced sampling and free energy calculations, apart from Figure S1a (Supporting Information). Figure S1a (Supporting Information) is an ordinary MD process within 300 ps under 473 K as a comparison to the MetaD results. This total process is presented in Video S1 (Supporting Information). The luteolin molecules tended to agglomerate due to their hydrophobicity, yet they did not undergo decomposition without CV. Figure S1b–e (Supporting Information) illustrates the results of the MetaD process at 423, 473, 523, and 573 K, respectively. At 423 and 473 K, the molecules aggregated within 40 ps, which is the same as Figure S1a (Supporting Information). The process of 473 K is presented in Video S2 (Supporting Information). Then, the decomposition occurred within 80 ps, and the devastation occurred within 200 ps. This process was consistent with the decomposition of organic molecules, producing carbon‐based fragments that could form graphitic carbon structures with hydrocarbon branches through the processes of polymerization, carbonization, as well as aromatization at the end under the SPRINT method that was identified as the CV, as demonstrated in Figure 1c.^[^ ^58^ ^]^ At 523 and 573 K, decomposition occurred before agglomeration, especially at 573 K, where decomposition occurred within 20 ps. These results indicated that the material underwent a drastic decomposition at these temperatures, as presented in Figure S1d,e (Supporting Information). The outcomes of such results did not meet the goal of constructing CPDs composed of the bioactive moieties of luteolin.

However, the analyses of captured snapshots during the simulations were not distinct enough. Thus, the free energies as a function of the CN have also been calculated by MetaD as presented in Figure 1d–g. As time increases, the agglomeration could be observed at 40 ps at 423 and 473 K in terms of the increase of CNs, as shown in Figure 1d,e. Then, the decrease in CNs illustrated the occurrence of decomposition. As shown in Figure 1f, the decline in CNs also indicates the process of decomposition. The arrow between 40 and 80 ps indicated a significant increase in the rate of decomposition during this interval. The subsequent increase in CNs suggested the nucleation of the fragments. As for 573 K in Figure 1g, the decomposition occurred at the beginning, while the nucleation process was simultaneously underway, resulting in a continued increase in CN. Collectively, at 523 and 573 K, the decomposing reaction was so drastic that too many luteolin molecules were depleted. The reaction at 423 K was not as effective as compared with that at 473 K, according to the larger CN at 423 K after 200 ps of simulation. Based on the above analysis, the temperature of 473 K is suitable for the predicted reaction. Hence, the synthesis of Lu‐CDs is then carried out under the guidance of the above reaction process.

**Figure 2:**
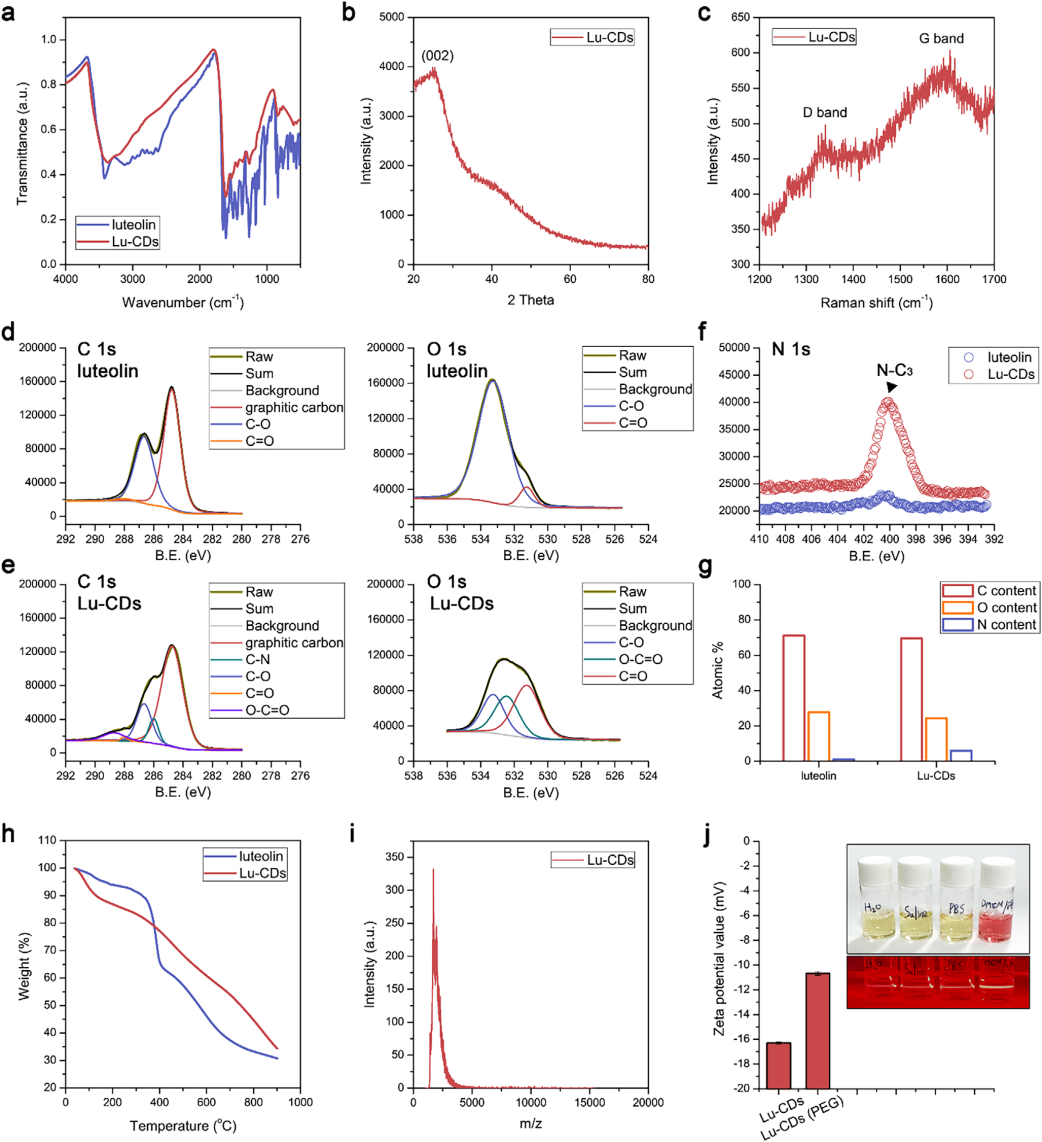
Characterization of Lu‐CDs. a) FT‐IR spectra of luteolin and Lu‐CDs. PXRD pattern b) and Raman spectrum c) of Lu‐CDs. High‐resolution C 1s and O 1s XPS spectra of luteolin d) and Lu‐CDs e). f) High‐resolution N 1s XPS spectra of luteolin and Lu‐CDs. g) The atomic percentages of carbon, oxygen, and nitrogen in luteolin and Lu‐CDs as evaluated through the deconvolution of the XPS spectra. h) TGA curves of luteolin and Lu‐CDs. i) MALDI‐TOF MS spectrum of Lu‐CDs. j) Zeta potential values of Lu‐CDs and PEGylated Lu‐CDs. Inset: digital photograph of Lu‐CDs dispersed in various media and the corresponding digital photograph for Tyndall effects. From left to right: water, saline, PBS, and DMEM containing 10% FBS. Data represent mean ± standard deviation (n = 3).

The Lu‐CDs were thus synthesized via a hydrothermal approach by using luteolin as the pharmacophoric precursor at 473 K in the presence of NH_3_·H_2_O. Such a carbonization process effectively converted the light yellow‐colored luteolin into a dark brown‐colored Lu‐CDs product (Figure S2, Supporting Information). Transmission electron microscopy (TEM) imaging, as depicted in Figures S3 and S4 (Supporting Information), indicated that these Lu‐CDs were extremely homogeneous, with an average size of 1.95 nm. Fourier transform infrared spectroscopy (FT‐IR) indicated the preservation of the major functional groups of luteolin on Lu‐CDs (**Figure** **2**a). As shown in Figure 2b,c, the characteristic diffraction in the powder X‐ray diffraction (PXRD) pattern and the D/G bands in the Raman spectrum of Lu‐CDs indicated the formation of the graphitic carbon cores. The high‐resolution TEM image in Figure S3d,e (Supporting Information) verified that Lu‐CDs exhibited fine crystallinity with a lattice spacing of 0.21 nm, attributed to the representative diffraction planes of the graphitic carbon. As shown in Figure 2d,e, high‐resolution X‐ray photoelectron spectroscopy (XPS) spectra in the C 1s and O 1s regions indicated the presence of C═O and C─O species on luteolin. After the carbonization process, the as‐prepared Lu‐CDs were covered with C═O, C─O, C─N, and O─C═O species. The appearance of O─C═O species on Lu‐CDs could be attributed to carbonization‐induced oxidation. As shown in Figure 2f, the NH_3_·H_2_O‐assisted hydrothermal process led to the formation of the nitrogen‐doped graphitic carbon cores containing N‐C_3_ species. As illustrated in Figure 2g, in comparison with the precursor luteolin, the increased nitrogen content and the decreased oxygen content could be ascribed to the processes of polymerization, carbonization, and aromatization that occur during the formation of the nitrogen‐doped graphitic carbon cores. Moreover, in comparison with luteolin, the significant changes in the thermogravimetric analysis (TGA) and derivative thermogravimetric analysis (DTG) curves demonstrated the newly formed Lu‐CDs (Figure 2h; Figure S5, Supporting Information). The molecular weight distribution of Lu‐CDs is in ≈1350–5000 as recorded by the matrix‐assisted laser desorption/ionization time‐of‐flight mass spectrometry (MALDI‐TOF MS) in Figure 2i.

**Figure 3:**
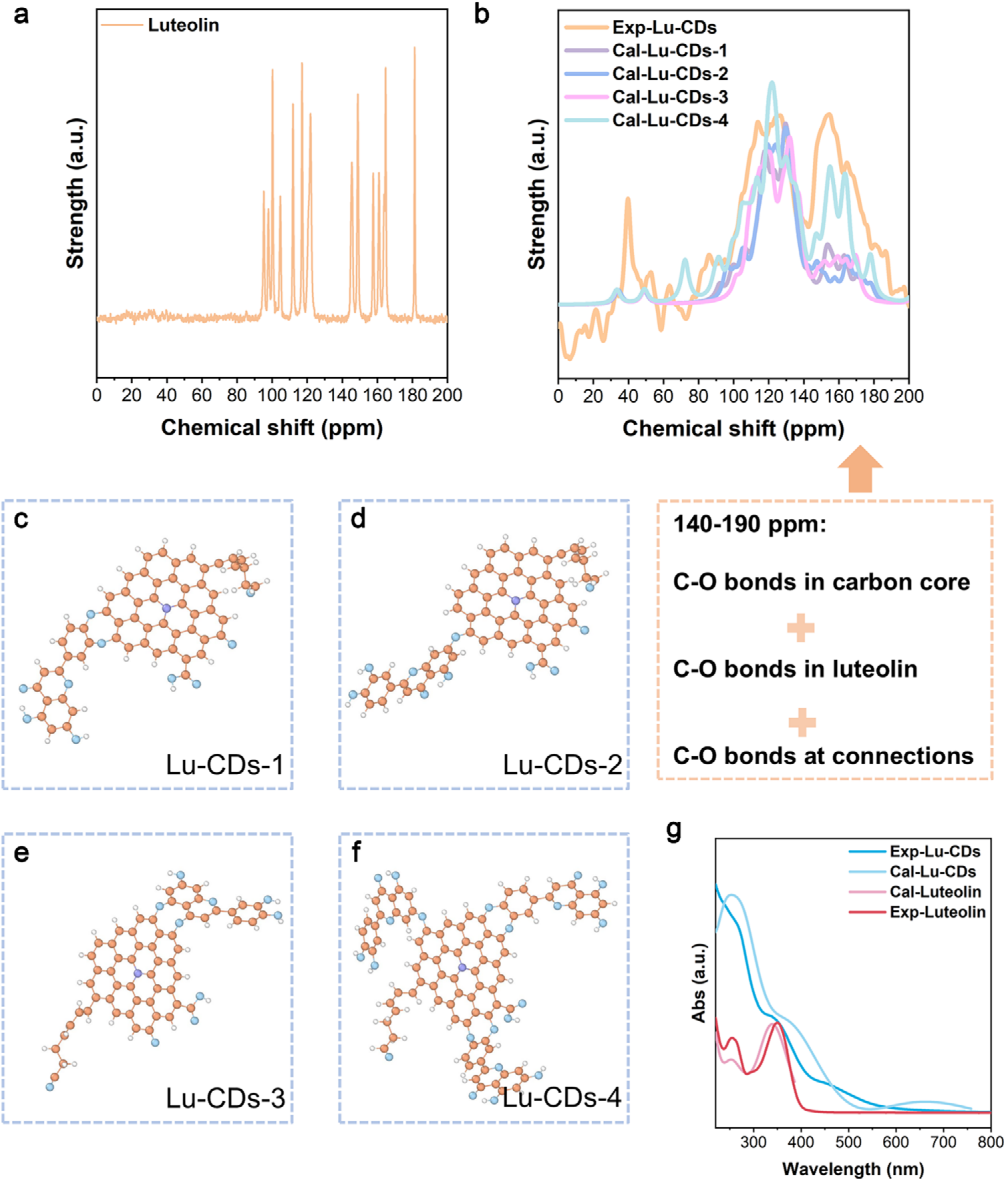
DFT calculations. a) The solid‐state ^13^C NMR spectrum of the luteolin molecule. b) The solid‐state ^13^C NMR spectrum of Lu‐CDs and the calculated ^13^C NMR spectra of Lu‐CDs are based on the DFT method. The experimental spectrum has been adjusted by +6.5 ppm to facilitate the comparison with the calculated spectra. c–f) The models of luteolin moieties‐conjugated carbon cores for NMR calculations, and light blue, orange, white, and light violet represent the O, C, H, and N, respectively. g) The UV–vis spectra contain both the experimental results and the calculated results.

To deeply understand the Lu‐CDs’ structure, DFT calculations were carried out. The calculated results of nuclear magnetic resonance spectroscopy (NMR) and UV–vis spectroscopy were supported by Multiwfn, as shown in **Figure** **3**, which were further compared with the experimental NMR results characterized by the cross‐polarization/magic angle spinning (CP/MAS) ^13^C NMR spectroscopy as well as the experimental UV–vis results.^[^ ^59^ ^]^ Figure 3a is the experimental ^13^C NMR result for luteolin, which is consistent with that reported previously.^[^ ^60^ ^]^ Figure 3b is the experimental and calculated ^13^C NMR results for the Lu‐CDs with broader peaks that are caused not only by the solid‐state NMR technology but also by the different bonding modes of the Lu‐CDs. In other words, there are many structural variables, including the sizes and oxidation states of the carbon cores, as well as the connection modes between the carbon cores and the bioactive moieties of luteolin, collectively resulting in the broadened NMR peaks. Thus, the full‐width at half‐maximum of the calculated ^13^C NMR for Lu‐CDs was set as 5 ppm. In comparison with luteolin, the significant changes in the experimental ^13^C NMR results demonstrated the newly formed Lu‐CDs. Based on the molecular dynamics (MD) processes, our deduction of the final structures for Lu‐CDs was tested. As shown in Figure 3b, the calculated NMR results for Lu‐CDs with four different types of connection modes between the carbon cores and the bioactive moieties of luteolin have been presented. Figure 3c–e are the Lu‐CDs that are composed of a single conjugated luteolin molecule. The single luteolin molecule was conjugated with the carbon cores through the O atoms after dehydrogenation in an alkaline environment. These connection sites are also the sites where luteolin can interact with some metal ions. The doped nitrogen atom is caused by the existence of ammonia during the synthetic process. Notably, the nitrogen species in the carbon cores were mainly N‐C_3_ species as indicated by the XPS spectra shown in Figure 2f. Thus, the N‐C_3_ species were presented in the carbon core structure. Figure 3f is the structure containing the three structures above with different types of connection modes. Generally, the deviation between the DFT results and the experimental results is quite large based on such an ordinary method, whose details are presented in the method section. Yet, the accurate NMR calculations were not the goal of the present study, but rather of the analysis of the final structure of Lu‐CDs. The total deviation of the whole peaks with 6.5 ppm is also acceptable in NMR calculations. Hence, all the experimental peaks in Figure 3b were shifted by adding +6.5 ppm for a distinct comparison between the calculated NMR results and the experimental NMR results. The peaks below 100 ppm are the formed hydrocarbon branches on the carbon core, while the peaks for the carbons on the carbon cores or the conjugated bioactive moieties of luteolin are centered in the 100–200 ppm range. Particularly, the peaks in the range of 140–190 ppm are predominantly ascribed to the carbon of the bioactive moieties of luteolin. Indeed, the NMR peak intensities in this range for Lu‐CDs‐4, comprising three conjugated luteolin moieties, are significantly higher than those of other Lu‐CDs models containing only one luteolin moiety. Such results also indicated that the real Lu‐CDs were composed of several luteolin moieties on a single carbon core. As shown in Figure S6 (Supporting Information), the peaks in the range of 140–190 ppm originated from the C─O bonds on both carbon cores and conjugated luteolin moieties based on the present DFT results. UV–vis was also used to verify our hypothetical structure. The experimental results, as well as the calculated results, are presented in Figure 3g. The absorption peaks for luteolin were centered at 255, 355 nm, and the peak below 220 nm, whereas the absorption peaks for Lu‐CDs were centered at 251, 332 nm, and the peak below 220 nm, which were out of the testing range. Then, time‐dependent DFT (TDDFT) methods were employed for the absorption spectra calculation. Here, the B3LYP functional was used for luteolin.^[^ ^61^ ^]^ However, it was observed that the transition states of Lu‐CDs remained predominantly in high‐energy fields. Consequently, the PBE0 functional was appropriate to adopt. 150 transition states and 700 transition states of luteolin and Lu‐CDs‐1 were calculated, respectively (Figure 3g). The differences between the UV–vis spectra of luteolin and Lu‐CDs also demonstrate the newly formed Lu‐CDs. As expected, the calculated spectra followed our experimental results. All these results indicated the correctness of our hypothetical structure for Lu‐CDs.

**Figure 4:**
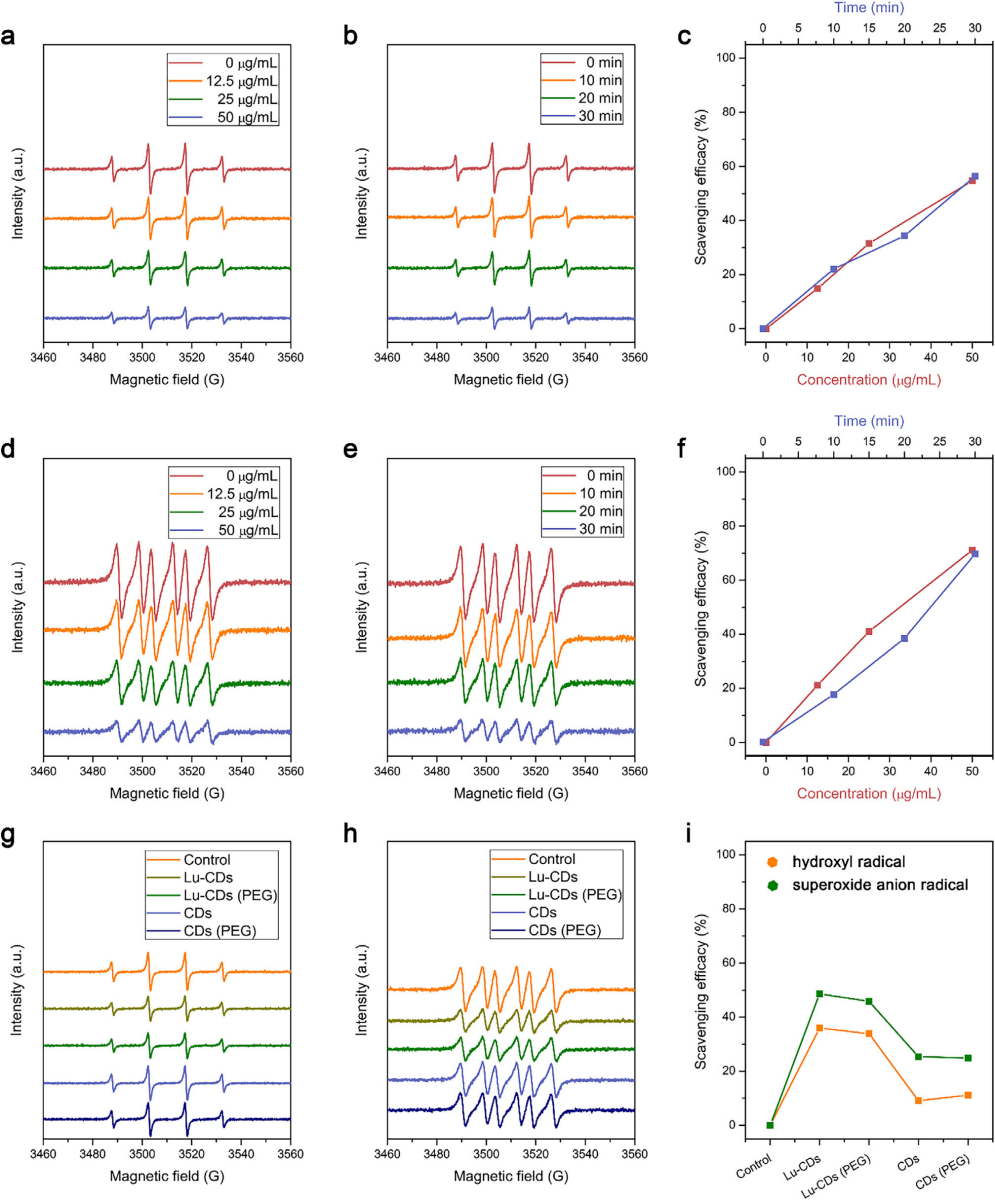
Radical scavenging activities of Lu‐CDs. Concentration‐dependent and time‐dependent radical scavenging activities of Lu‐CDs for OH· a,b) and ·O_2_^−^ d,e) as demonstrated by ESR spectra as well as the related quantitative analysis results c,f). For concentration‐dependent experiments, the reaction period is 30 min. For time‐dependent experiments, the concentration of Lu‐CDs is 50 µg mL^−1^. The radical scavenging activities for OH· g) and ·O_2_^−^ h) of Lu‐CDs, PEGylated Lu‐CDs, CDs, and PEGylated CDs as demonstrated by ESR spectra under the same experimental conditions. i) Comparison of the radical scavenging efficacies of Lu‐CDs, PEGylated Lu‐CDs, CDs, and PEGylated CDs as quantified by the ESR signal intensities under the same experimental conditions.

Considering the antioxidative luteolin moieties on Lu‐CDs, the antioxidative activity of Lu‐CDs was thus tested through the electron spin resonance (ESR) spectroscopy with DMPO as the spin trapping probe, which has been regarded as the most direct and reliable technology for the identification and quantification of short‐lived ROS. The efficacies of Lu‐CDs in scavenging hydroxyl radicals (OH·) and superoxide anion radicals (·O_2_^−^) were thus investigated. As shown in **Figure** **4**a–c, obviously decreased ESR signal intensities for the spin adduct of OH· could be observed in the presence of Lu‐CDs, suggesting the effective OH· scavenging activity of Lu‐CDs. Likewise, as shown in Figure 4d–f, significantly decreased ESR signal intensities for the spin adduct of ·O_2_^−^ could be observed in the presence of Lu‐CDs, indicating the effective ·O_2_^−^ scavenging activity of Lu‐CDs. The antioxidative activity for OH· and ·O_2_^−^ by Lu‐CDs was also concentration‐dependent and time‐dependent. For comparison, citric acid‐derived CPDs (CDs) composed of only graphitic carbon cores and oxygenated groups were prepared according to our previous study. As shown in Figure 4g–i, compared with CDs, significantly enhanced radical scavenging activities of Lu‐CDs toward both OH· and ·O_2_^−^ could be observed, which could be attributed to the enriched conjugated luteolin moieties on Lu‐CDs. For further biological usages, Lu‐CDs were noncovalently functionalized with polyethylene glycol (PEG) through the hydrophobic interaction between the graphitic carbon cores and the terminal phospholipid groups of DSPE‐mPEG to increase the biostability of Lu‐CDs. As shown in Figure 2j and Figure S7 (Supporting Information), the ζ potential value of Lu‐CDs after PEGylation increased compared with that of Lu‐CDs, from −16.30 ± 0.10 mV to −10.67 ± 0.15 mV. The photographs in Figure 2j and Figure S8 (Supporting Information) demonstrated that PEGylated Lu‐CDs showed high dispersibility in water, saline, phosphate‐buffered saline (PBS), and Dulbecco's modified Eagle medium (DMEM) containing 10% fetal bovine serum (FBS), and distinct Tyndall effects could be observed. As shown in Figure 4g–i, the post‐PEGylation process did not significantly influence the radical scavenging activity of Lu‐CDs. For convenience, the PEGylated Lu‐CDs were also termed Lu‐CDs in the following sections of biological studies.

**Figure 5:**
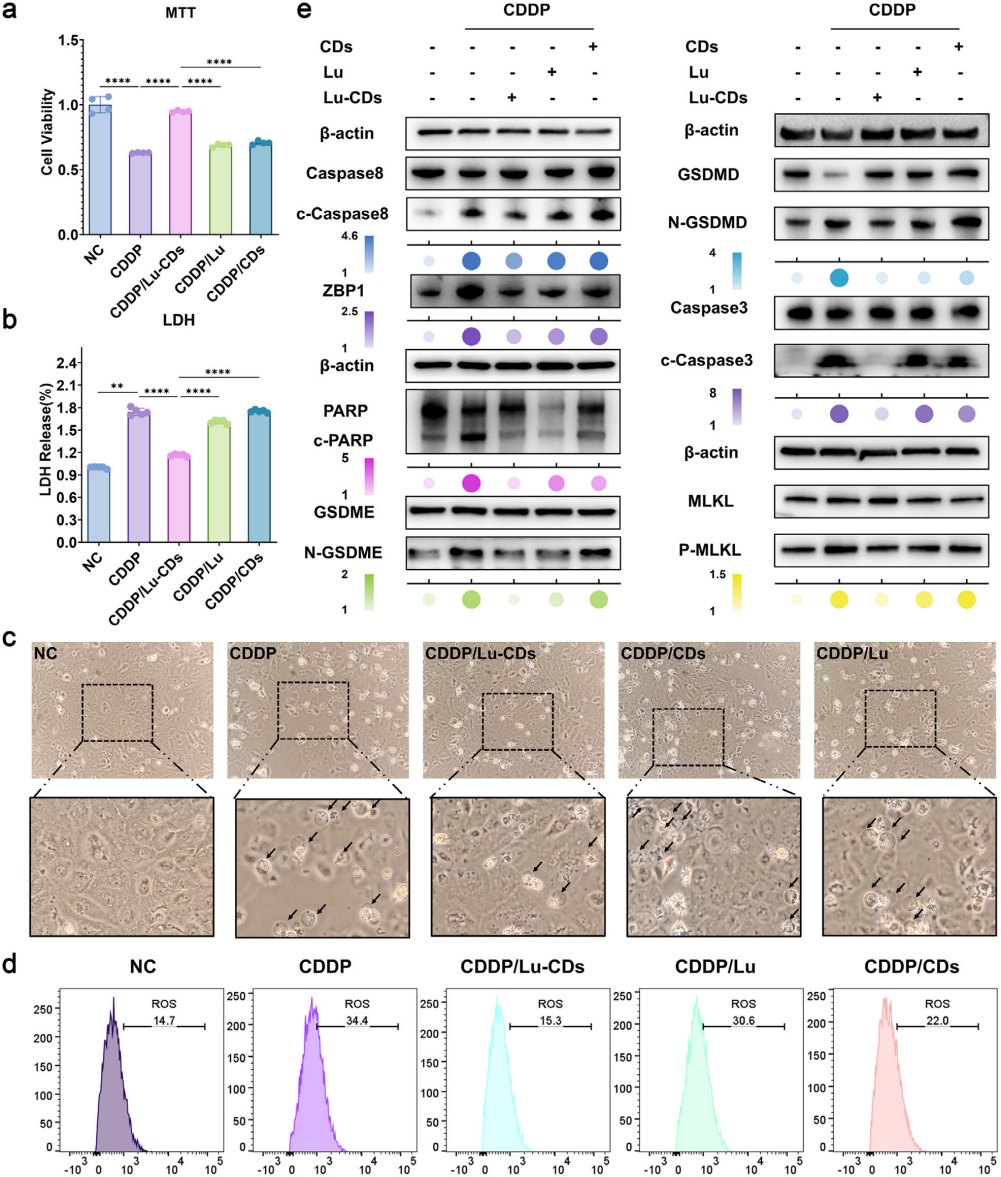
Lu‐CDs inhibit PANoptosis in vitro and relieve cisplatin‐induced cytotoxicity. a) Cell viability of HK‐2 cells exposed to cisplatin (CDDP) following treatment with Lu‐CDs, CDs, or luteolin (Lu), n = 4. b) LDH release in cisplatin‐treated HK‐2 cells after incubation with Lu‐CDs, CDs, or luteolin (n = 5). c) Typical bright‐field images illustrate the cellular morphology in various experimental groups. Black arrows indicate morphological changes associated with pyroptosis. d) Flow cytometry analysis of intracellular ROS levels using DCFH‐DA as a fluorescent probe. e) Western blot analysis of the biomarkers for PANoptosis in various experimental groups. The color intensity and size of the colored bubbles correspond to relative protein expression levels, with darker hues and larger diameters indicating higher expression. For in vitro studies, the Lu‐CDs or CDs used were at 25 µg mL^−1^, while luteolin was applied at its optimal concentration of 5 µM. Higher concentrations of luteolin induced significant cytotoxicity, whereas lower concentrations showed insufficient therapeutic efficacy. Data represent mean ± standard deviation. The Mann–Whitney test or one‐way analysis of variance (Dunnett's *t*‐test) was performed on the studied data. Asterisks indicate statistically significant differences (^*^ *p*< 0.05, ^**^ *p*< 0.01, ^***^ *p*< 0.001, and ^****^ *p*< 0.0001).

Next, we investigated the efficacy of Lu‐CDs for mitigating chemodrug‐induced nephrotoxicity in vitro on the human kidney‐2 (HK‐2) cells by using cisplatin as the model drug. The in vitro biosafety of Lu‐CDs was investigated first on HK‐2 cells and the human hepatic stellate cell line LX‐2. As shown in Figure S9 (Supporting Information), Lu‐CDs exhibited negligible cytotoxicity toward HK‐2 and LX‐2 cells even at elevated concentrations, indicating favorable biosafety. Then, HK‐2 cells were treated with cisplatin (15 µg mL^−1^). MTT assay was adopted to investigate the efficacy of Lu‐CDs to alleviate the cytotoxicity of cisplatin in HK‐2 cells. As depicted in **Figure** **5**a, cisplatin treatment significantly reduced the viability of HK‐2 cells, whereas CDs and luteolin slightly attenuated cisplatin‐induced cytotoxicity. Remarkably, Lu‐CDs could attenuate the cisplatin‐induced cytotoxicity most effectively. Live/dead staining images in Figure S10 (Supporting Information) also demonstrated the detoxification performance of Lu‐CDs. Compared with the precursor luteolin, the obviously higher detoxification activity of Lu‐CDs could be ascribed to the nanoformulation of Lu‐CDs. On the contrary, citric acid‐derived CDs composed of only graphitic carbon cores and oxygenated groups showed no obvious detoxification effects on the viability of cisplatin‐treated HK‐2 cells under identical experimental conditions, which could be attributed to the absence of pharmacophoric moieties from luteolin and the relatively lower antioxidative activity of CDs. Subsequently, the in vitro ROS scavenging capacity of Lu‐CDs was evaluated using flow cytometry with 2′,7′‐dichlorodihydrofluorescein diacetate (DCFH‐DA) as the fluorescent sensor. Cisplatin‐treated HK‐2 cells exhibited a significant increase in intracellular ROS levels. As expected, Lu‐CDs displayed the most potent ROS scavenging activity among various experimental groups (Figure 5d). As PANoptosis is a regulated cell death pathway with typical characteristics of pyroptosis, apoptosis, and necroptosis, we explored whether cisplatin could induce PANoptosis and cell death in HK‐2 cells. We first investigated the apoptosis‐inhibiting activity of Lu‐CDs in cisplatin‐treated HK‐2 cells. The Annexin V‐FITC apoptosis assay results demonstrated that Lu‐CDs showed the highest anti‐apoptotic activity in cisplatin‐treated HK‐2 cells, which could be attributed to their potent ROS scavenging capacity and nanoscale structure (Figure S11, Supporting Information). Concurrently, western blot analysis was employed to examine the expression of apoptosis‐related proteins. The expression levels of cleaved caspase 3 and cleaved PARP, the key apoptotic biomarkers, indicated that Lu‐CDs performed a high anti‐apoptotic activity in cisplatin‐treated HK‐2 cells (Figure 5e). Besides, pyroptosis represents another vital pathway during the induction of PANoptosis. The release of lactate dehydrogenase (LDH) can serve as a crucial biomarker of cell lysis that reflects the extent of pyroptosis. As shown in Figure 5b, cisplatin treatment induced a significant increase in the LDH release of HK‐2 cells. The luteolin could also attenuate cisplatin‐induced LDH release owing to its pyroptosis‐inhibiting activity. It's noteworthy that Lu‐CDs demonstrated the most effectiveness in inhibiting LDH release, whereas CDs showed a negligible inhibitory effect on LDH release under the same experimental conditions. Compared with CDs with solely antioxidative activity, the desirable pyroptosis‐inhibiting activity of Lu‐CDs could be ascribed to the enriched pharmacologically active moieties from luteolin for pyroptosis inhibition, as well as the higher radical scavenging activity. The pyroptotic morphology of cisplatin‐treated HK‐2 cells following different treatments was further investigated using optical microscopy. As shown in Figure 5c, cisplatin exposure induced pyroptotic membrane blebbing obviously in HK‐2 cells. Strikingly, treatment with Lu‐CDs significantly reversed these morphological alterations of pyroptosis. Western blot analysis was used to explore the expression of pyroptosis‐associated proteins, including GSDMD‐N, GSDME‐N, and caspase 8. Indeed, similar trends could be observed, as shown in Figure 5e. In addition to apoptosis and pyroptosis, we investigate the necroptosis‐inhibiting activity of Lu‐CDs. The expression levels of necroptosis‐associated proteins, including ZBP1 and the phosphorylation of MLKL, were thus investigated. As shown in Figure 5e, western blot results indicated that Lu‐CDs could effectively relieve cisplatin‐induced necroptosis in HK‐2 cells. On the contrary, both luteolin and CDs showed a poor positive effect on necroptosis inhibition. All the above results indicated that Lu‐CDs with enriched pharmacophoric moieties of luteolin could effectively inhibit PANoptosis and mitigate cisplatin‐induced cytotoxicity in vitro. Notably, Lu‐CDs demonstrated significantly stronger PANoptosis inhibition compared to free luteolin, indicating that Lu‐CDs not only retain the bioactivity of luteolin but also exhibit enhanced potency against PANoptosis.

**Figure 6:**
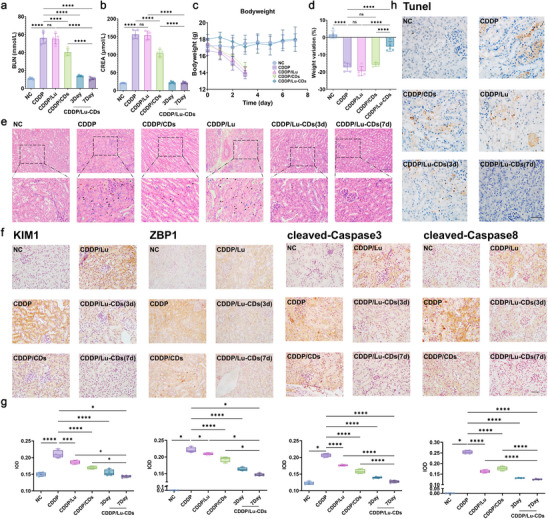
Lu‐CDs inhibit PANoptosis in vivo and relieve AKI. Blood urea nitrogen levels a) and serum creatinine levels b) of AKI mice in various experimental groups on day 3 (NC, CDDP, CDDP/Lu, CDDP/CDs, CDDP/Lu‐CDs) and day 7 (CDDP/Lu‐CDs) post‐AKI induction (n = 5). c) Time‐dependent bodyweight changes of AKI mice following different treatments (n = 5). d) Bodyweight changes of AKI mice after various treatments on day 3 post‐AKI induction (n = 5). e) Representative H&E‐stained renal tissue sections showing casts (black plus symbols), granulovacuolar degeneration of renal tubular epithelial cells (black arrows), detachment of renal tubular epithelial cells (red arrows), tubular dilation (green plus symbols), and immune cell infiltration (blue arrows) on day 3 (NC, CDDP, CDDP/Lu, CDDP/CDs, CDDP/Lu‐CDs) and day 7 (CDDP/Lu‐CDs) post‐AKI induction. The scale bar is equal to 100 µm. f) Representative IHC staining images of kidney sections. The scale bar is equal to 50 µm. g) Quantitative analysis of IHC staining images (n = 4). h) TUNEL assay of kidney sections demonstrating apoptotic cells. The scale bar is equal to 50 µm. Data represent mean ± standard deviation. The Mann–Whitney test or one‐way analysis of variance (Dunnett's *t*‐test) was performed on the studied data. Asterisks indicate statistically significant differences (^*^ *p*< 0.05, ^**^ *p*< 0.01, ^***^ *p*< 0.001, and ^****^ *p*< 0.0001). NC indicates the negative control group (healthy mice).

Before exploring the efficacy of Lu‐CDs in AKI mitigation, the biocompatibility was assessed in detail. A hemolytic assay examined the interaction between Lu‐CDs and blood components. Figures S12 and S13 (Supporting Information) show no significant hemolysis following co‐incubation with Lu‐CDs. Histopathological analysis using hematoxylin and eosin (H&E) staining of major organs, including the heart, liver, spleen, lung, and kidney from mice after intravenous injection of Lu‐CDs revealed no evidence of systemic toxicity, demonstrating the high biocompatibility of Lu‐CDs (Figure S14, Supporting Information). Furthermore, bodyweight variation measurements in the test group showed negligible differences compared to the control group (Figure S15, Supporting Information). Additionally, blood biochemistry and hematology analyses demonstrated no obvious differences between the test and control groups (Figures S16–S20, Supporting Information). The biodistribution of Lu‐CDs was then investigated in a murine model of cisplatin‐induced AKI. Healthy Balb/c mice were injected with cisplatin (15 mg kg^−1^) intraperitoneally. The time point at 24 h post‐injection of cisplatin was defined as the initiation of AKI. To visualize the biodistribution of Lu‐CDs in AKI mice, Cy5‐labeled Lu‐CDs (Lu‐CDs‐Cy5) were used for time‐dependent ex vivo fluorescence imaging. As displayed in Figure S21 (Supporting Information), a significant accumulation of Lu‐CDs‐Cy5 in the injured kidneys could be observed in AKI mice after intravenous injection. Particularly, the fluorescence signals from Lu‐CDs‐Cy5 could persist in the kidneys up to 72 h post‐injection, indicating the prolonged circulation time and renal retention of Lu‐CDs, which could be further verified by the quantitative results (Figure S22, Supporting Information). The above results collectively demonstrate the in vivo safety profile and renal accumulation properties of Lu‐CDs, highlighting their potential for treating AKI. The feasibility of Lu‐CDs for mitigating chemodrug‐induced nephrotoxicity in vivo was thus investigated in a murine model of cisplatin‐induced AKI. Mice were divided into 5 groups randomly, including the negative control (mice + saline), the positive control (AKI mice + saline), AKI mice with treatment of luteolin (4 mg kg^−1^), AKI mice with treatment of CDs (4 mg CDs kg^−1^), and AKI mice with treatment of Lu‐CDs (4 mg Lu‐CDs kg^−1^). Elevated serum creatinine (CREA) and blood urea nitrogen (BUN) levels are well‐established clinical manifestations to assess renal function, reflecting the accumulation of nitrogen metabolism end‐products. As shown in **Figure** **6**a,b, AKI mice showed significantly increased CREA and BUN levels. Luteolin treatment exhibited a negligible therapeutic effect on AKI, whereas CDs treatment could partially restore the renal function of AKI mice. Crucially, Lu‐CDs could achieve near‐complete restoration of renal function by both day 3 and day 7 post‐induction of AKI. Besides, the measurements of the bodyweight variation of AKI mice in various experimental groups further demonstrated the treatment benefits, with all the AKI mice in the Lu‐CDs treatment group surviving on day 7 post‐induction of AKI (Figure 6c,d). Notably, the AKI mice in the Lu‐CDs treatment group showed no detectable differences in bodyweight variation compared with the control healthy mice on day 7 post‐induction of AKI (Figure S23, Supporting Information). Furthermore, hematoxylin and eosin (H&E) staining was performed to conduct the histopathological analysis of kidneys from mice in various experimental groups. As displayed in Figure 6e, the renal tubule damage, as well as the formation of casts, the hallmark of AKI, could be observed in a large number of the kidney tissues from AKI mice with the treatment of saline or luteolin. In contrast, obviously fewer damaged structures could be observed in the kidney tissues from AKI mice with the treatment of CDs. Importantly, the kidney tissues from AKI mice treated with Lu‐CDs showed no obvious structural damage on both day 3 and day 7 post‐induction of AKI. All these in vivo experimental results indicated that Lu‐CDs were the most effective in mitigating cisplatin‐induced nephrotoxicity, which could be attributed to the high renal accumulation, the long body retention, and the high PANoptosis‐inhibiting activity of Lu‐CDs. By contrast, the lack of required targeting ability or PANoptosis‐inhibiting activity made the precursor luteolin and nanoscale CDs show insufficient therapeutic efficacy for AKI.

Going further, immunohistochemistry (IHC) staining was used to investigate the underlying mechanisms of Lu‐CDs in mitigating cisplatin‐induced nephrotoxicity in vivo. As expected, cisplatin treatment caused significantly increased expressions of PANoptosis‐related biomarkers involving cleaved caspase‐3, cleaved caspase‐8, and ZBP1 in the kidneys of AKI mice. Luteolin treatment showed a moderate effect on the expression of the above biomarkers, whereas CDs treatment could also partially decrease the expression of cleaved caspase‐3, cleaved caspase‐8, and ZBP1 in the kidneys of AKI mice owing to the kidney retention properties of CDs. As expected, Lu‐CDs exhibited a significant PANoptosis inhibitory effect on cisplatin‐induced nephrotoxicity in vivo, as verified by the IHC staining images in Figure 6f,g. Terminal deoxynucleotidyl transferase‐mediated dUTP nick‐end labeling (TUNEL) staining was further conducted to explore the therapeutic effect of Lu‐CDs for AKI. As shown in Figure 6h and Figure S24 (Supporting Information), Lu‐CDs showed the highest apoptosis‐inhibiting activity among all experimental groups. Besides, the renal expression of kidney injury molecule‐1 (KIM‐1) was also tested by IHC staining to evaluate the degree of kidney injury. Indeed, the kidney slices from AKI mice had the highest level of KIM‐1, and Lu‐CDs treatment could effectively relieve the kidney injury (Figure 6f,g). Importantly, on day 7 post‐induction of AKI, all the above biomarkers indicated that Lu‐CDs could completely recover the pathological injury in the kidneys of AKI mice.

**Figure 7:**
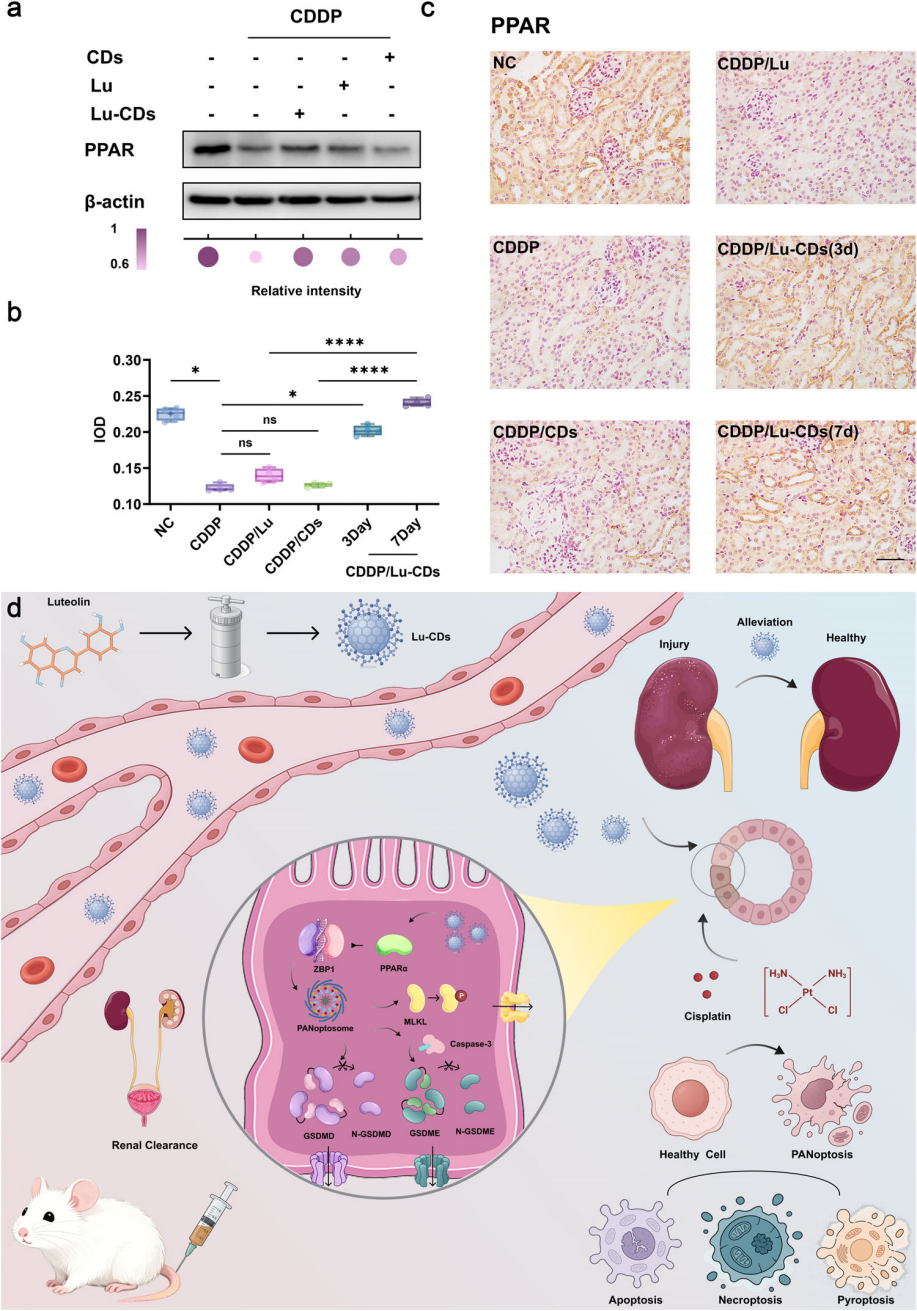
Lu‐CDs activate the expression of PPARα. a) Western blot analysis of the expression of PPARα in various experimental groups. The color intensity and size of the colored bubbles correspond to relative protein expression levels, with darker hues and larger diameters indicating higher expression. b) Quantitative analysis of IHC staining images (n = 4). c) Representative IHC staining images of kidney sections. The scale bar is equal to 50 µm. d) Schematic illustration of the synthesis and application of Lu‐CDs for PANoptosis inhibition and the alleviation of chemodrug‐induced nephrotoxicity. Data represent mean ± standard deviation. The Mann–Whitney test or one‐way analysis of variance (Dunnett's *t*‐test) was performed on the studied data. Asterisks indicate statistically significant differences (^*^ *p*< 0.05, ^**^ *p*< 0.01, ^***^ *p*< 0.001, and ^****^ *p*< 0.0001). Image adapted from Servier Medical Art (https://smart.servier.com/), licensed under CC BY 4.0 (https://creativecommons.org/licenses/by/4.0/).

Considering the vital role that PPARα plays in AKI protection, we investigated the expression of PPARα both in vitro and in vivo to explore the potential biological mechanism for Lu‐CDs for PANoptosis inhibition. As shown in **Figure** **7**a, consistent with expectations, PPARα expression in cisplatin‐treated HK‐2 cells was significantly downregulated relative to the control group. While CDs possessing solely antioxidative activity marginally upregulated PPARα expression, the small molecule luteolin demonstrated pharmacological activation of PPARα in cisplatin‐treated cells. Notably, Lu‐CDs exhibited remarkably enhanced PPARα activation efficacy compared to the precursor, luteolin. The expression of PPARα in the kidneys from various groups in the in vivo experiments was further investigated by IHC staining. As shown in Figure 7b,c, the kidney slices from AKI mice treated with saline or CDs had the lowest level of PPARα expression. In contrast, luteolin could activate the PPARα expression to some extent, and Lu‐CDs treatment could upregulate the expression of PPARα most effectively. These results collectively indicated that Lu‐CDs with enriched pharmacophoric moieties of luteolin could effectively inhibit PANoptosis via activating PPARα and mitigate cisplatin‐induced nephrotoxicity in vivo. These results indicate that Lu‐CDs inherit the bioactivity of luteolin, implicating PPARα activation as a potential mechanism underlying their PANoptosis‐inhibitory effects (Figure 7d).

To further elucidate the action mode of Lu‐CDs, a panel of pharmacological inhibitors—including the apoptosis inhibitor Z‐IETD‐FMK, the necroptosis inhibitor Necrostatin‐1, and the NLRP3 inhibitor MCC950 (to block pyroptosis)‐ was applied for mechanistic investigations. As shown in Figure S25 (Supporting Information), individual blockade of apoptosis, necroptosis, or pyroptosis did not reduce the protective effect of Lu‐CDs against cisplatin‐induced cytotoxicity in HK‐2 cells, as evidenced by cell viability assays and live/dead staining. These results confirm that Lu‐CDs can concurrently suppress apoptosis, pyroptosis, and necroptosis, supporting their role as a potent PANoptosis inhibitor. Our findings have indicated that Lu‐CDs could effectively activate PPARα and inhibit PANoptosis. In contrast, structurally analogous non‐luteolin carbonized polymer dots, which exhibit only antioxidative activity and lack the pharmacophoric moieties of luteolin, showed minimal PPARα activation and no significant PANoptosis‐inhibiting activity. We further conducted experiments to verify whether the pharmacological activity of Lu‐CDs originates from the surface‐retained pharmacophoric moieties of luteolin. To this end, we introduced an additional control group consisting of a physical mixture of CDs and free luteolin, in order to evaluate the structural essentiality of the surface‐presented luteolin moieties in Lu‐CDs. The concentration of free luteolin was used at a pharmacologically active but non‐cytotoxic level, while the concentration of CDs was matched to that of Lu‐CDs. As shown in Figure S26 (Supporting Information), we assessed PANoptosis biomarker expression through a series of assays, including Western blot analysis of key proteins, along with cell viability assays, and cellular morphology observation. The results clearly demonstrated that the physical mixture of CDs and free luteolin failed to inhibit PANoptosis or alleviate cisplatin‐induced cytotoxicity in HK‐2 cells to an extent comparable with Lu‐CDs. Together, these findings strongly support the conclusion that the bioactivity of Lu‐CDs critically depends on the functional presentation of luteolin‐derived pharmacophoric moieties on their surface.

While this study provides compelling evidence for the renoprotective effects of Lu‐CDs, we acknowledge its limitations, particularly concerning the comprehensive safety and biodistribution profile. A critical evaluation of potential off‐target effects, long‐term biosafety, and biodistribution beyond the kidneys is indeed essential for clinical translation. Our future work will quantitatively track the biodistribution of Lu‐CDs in major organs beyond the kidneys, including the heart, liver, spleen, and lungs, over extended periods. Comprehensive toxicological studies will be conducted to evaluate potential off‐target effects and long‐term safety, focusing on immune responses, hematological parameters, and histological changes of vital organs. These investigations are crucial for guiding the rational development of Lu‐CDs toward potential therapeutic applications.

## 3 Conclusion

In summary, we have developed an enhanced sampling method for MD using trained ML‐FF to guide the rational design of bioactive Lu‐CDs incorporating specific pharmacophoric moieties derived from flavonoid compound luteolin. Unlike conventional antioxidative nanocarbon‐based therapeutics, these Lu‐CDs demonstrated dual functionality, combining radical scavenging with PANoptosis‐inhibiting activity. In a murine model of chemodrug‐induced AKI, Lu‐CDs administered at a very low dose of 4 mg kg^−1^ exhibited prolonged renal accumulation of over 72 h and elicited superior nephroprotective efficacy compared to both the parent small‐molecule luteolin and antioxidative CPDs with solely radical scavenging activity, which could effectively restore the renal function of the injured kidneys by blocking PANoptosis through activating the expression of PPARα. These findings not only establish a computer‐assisted design strategy for bioactive nanodrugs but also identify PANoptosis inhibition as a promising therapeutic avenue for mitigating chemodrug‐induced nephrotoxicity. Our primary objective in the present study is to provide a mechanistic proof‐of‐concept, specifically focusing on chemodrug‐induced nephrotoxicity—a major dose‐limiting side effect in chemotherapy. As the cisplatin‐induced AKI model is well‐established and widely used for assessing the potential of nanodrugs in mitigating nephrotoxicity, we therefore selected it to systematically investigate the proposed mechanism of Lu‐CDs, namely, the inhibition of PANoptosis. We believe this focused and in‐depth exploration within a clinically relevant context offers valuable insights and establishes a solid foundation for subsequent research. Future studies involving other nephrotoxic chemotherapeutic agents (e.g., methotrexate) or different kidney injury models (e.g., ischemia‐reperfusion) will be essential to fully validate the therapeutic potential and generalizability of Lu‐CDs.

## 4 Experimental Section

### Animals

Female Balb/c mice (6–8 weeks old) and female Kunming mice (6–8 weeks old) were obtained from Charles River Laboratories. All animal procedures conducted in this study were approved by the Ethics Committee of Beijing Institute of Pharmacology and Toxicology (Approval No.: IACUC‐DWZX‐2025‐P565). Female Balb/c mice were utilized to assess the biosafety of Lu‐CDs and their therapeutic efficacy in treating AKI, while female Kunming mice were employed for hemolysis testing of Lu‐CDs. All animals were handled in compliance with relevant ethical guidelines. The mice were housed under controlled environmental conditions (temperature: 20–26 °C; light/dark cycle: 12 h/12 h) with ad libitum access to food and water. After a one‐week acclimatization period, the animals were randomly allocated to experimental groups. All subsequent data collection and analyses were performed by investigators blinded to group assignments. At the end of the treatment period, laboratory personnel euthanized the animals in strict adherence to institutional animal ethics protocols.

### Statistical Analysis

All data were expressed as mean ± standard deviation (SD) and were obtained from at least 3 specimens. The GraphPad 6.0 software was used to analyze experimental data. The Mann–Whitney test or one‐way analysis of variance (Dunnett's *t*‐test) was performed on the studied data. A *p*‐value<0.05 was considered statistically significant. Asterisks indicated significant differences (^*^ *p*< 0.05, ^**^ *p*< 0.01, ^***^ *p*< 0.001, ^****^ *p*< 0.0001).

## Conflict of Interest

The authors declare no conflicts of interest.

## Supporting information



Supporting Information

Supplemental Video 1

Supplemental Video 2

## Data Availability

The data that support the findings of this study are available from the corresponding author upon reasonable request.
