# Supplementary material for: Construction of PANoptosis‐Inhibiting Carbonized Polymer Dots via Machine Learning Potential for Mitigating Chemodrug‐Induced Nephrotoxicity
Source: Adv Sci (Weinh). 2025 Dec 12;13(12):e12196. doi: 10.1002/advs.202512196 (PMC12948267; doi:10.1002/advs.202512196)
Supplement: Supplementary file 1 — Supporting Information [file ADVS-13-e12196-s001.docx]

Supporting Information

Construction of PANoptosis-Inhibiting Carbonized Polymer Dots via Machine Learning Potential for Mitigating Chemodrug-Induced Nephrotoxicity

Xinchen Liu, Jiaxin Zhang, Xiangyu Yan,* Nuo Li, Yu-Chao Dong, Zihao Wang,* Daowei Li,* Yong Du, Huan Wang*

**Ⅰ. Methods**

**DFT configurations for ML-FF.**

In this work, machine learning was employed to develop a machine learning force field (ML-FF) for carrying out molecular dynamics simulations of the hydrothermal synthesis process of Lu-CDs. Conventional empirical force fields are unable to reliably describe the complex chemical reactions and bond formation/breaking events involved in this process. The ML-FF enabled us to simulate the reaction pathway and intermediate structures in a way that standard methods cannot, providing atomic-level insight into the formation mechanism of Lu-CDs. This application of machine learning is different from its more common use in predicting material properties or guiding experimental design. Here, it served as a foundational tool to model the synthesis process itself, which was central to understanding how the structural and functional features of Lu-CDs arise.

The Vienna ab initio package (VASP) was employed for AIMD to generate a training database encompassing a range of temperatures, including 273K, 423K, 473 K, 523 K, 723 K, and 1273 K. The functional was selected as Perdew-Burke-Ernzerhof (PBE), while the projector-augmented-wave potential (PAW) was set as the pseudopotential.^[1]^ The cut-off energy of the plane wave was 400 eV in the AIMD process. The k-point spacing was 2π×0.03 Å^-1^, and the converged criteria of the self-consistent field (SCF) was 10^-5^ eV. Considering the weak interactions, we included the vdW correction by the DFT-D3(BJ) method.^[2]^ In the AIMD, the timestep was set to 1 fs under the velocity-rescaled thermostat with the NVT ensemble. Each temperature comprised 20,000 steps, with configurations selected per 100 steps to form the final training database. All the molecular images were generated by OVITO software.^[3]^

The FF was generated by the DeepMD-kit package by neuron network learning.^[4]^ The cut-off for searching the neighbor atoms was set as 6.0 Å, and the smoothing function started from 0.5 Å. The three sizes of hidden layers were set as 25, 50, and 100, with 120 neurons of the fitting net in each layer. At the beginning, the learning rate was selected as 0.001 decaying every 5000 steps, while the 3.51×10^-8^ was set at the end. The training processes lasted 1000000 steps to obtain better results and avoid overfitting results. The training database contained the AIMD configurations, including 273K, 423K, 473 K, 523 K, 723 K, and 1273 K temperature results.

**MetaD and MD simulations.**

MetaD and MD for luteolin decomposition were carried out by LAMMPS based on the training FF. To prevent the O atoms from anomalously converging in the simulation processes, Lennard-Jones potential with 0.00884 eV and 3.480 Å of ε and σ was added, respectively.^[5, 6]^ The NVT ensemble was adopted for these simulations at different temperatures. The timestep was 1 fs. For the MetaD simulations,^[7]^ the CV was defined as

$CN=\frac{1-\left(r/r_{0}\right)^{n}}{1-\left(r/r_{0}\right)^{m}}$ (1)

Here *r* is the distance between each atom, $r_{0}$ (C-C 1.42 Å) is the standard distance between each atom. m and n were defined as 12 and 6, respectively. The CV was added at every 20 steps of the MD processes, and the Gaussian height and width of the CV were set as 0.01 eV and 0.1, respectively.

The nucleation processes were generated by CP2K (v2023.1), which incorporated PLUMED for MetaD simulations.^[8]^ The plane-wave cut-off was set to 600 Ry, and the mixed basis set employed was DZVP-MOLOPT-SR-GTH. The convergence criterion for the SCF was set to 10^-5^ Ry. The selected functional was Geometry, Frequency, and Noncovalent Interaction Extended Tight Binding (GFN-xTB) by Grimme et al..^[9]^ The time step of these simulations was set to 1 fs at 473 K, the temperature at which our hydrothermal reaction occurred. To simulate the nucleation process, we did a total of 300 ps simulations of the MetaD process. The CVs were selected as SPRINT,^[10]^ which was defined as

$s_{i}=\sqrt{N}\lambda v_{i}$ (2)

In Equation (2), $N$ is the total number of atoms, $\lambda$ and $v_{i}$ represent the eigenvalue and eigenvector in the contact matrix between different atoms, $\lambda$ is selected as the largest eigenvalue while $v_{i}$ is the largest eigenvector in the calculation. The N×N matrix consists of all possible distances between atoms. The height and width of Gaussian hills in stage 2 are 0.1 eV and 0.2 respectively at every 50 molecular dynamics steps.

**NMR and UV-Vis spectra calculations.**

We used the Gaussian 16 Rev. C 01 to calculate the NMR and UV-Vis absorption spectra.^[11]^ The NMR calculation was based on the gauge-independent atomic orbital (GIAO) methodology.^[12]^ The geometric optimization was carried out based on B3LYP functional with 6-31g(d) basis set at the gas phase. Then the NMR calculations were based on the revTPSS functional with pcSseg-1 basis set using the SMD model for chloroform.^[13, 14]^ The chemical shift,

$\delta_{i}=\sigma_{ref}-\sigma_{i}+\delta_{ref}$ (3)

Where the $\sigma_{ref}$ and $\sigma_{i}$ are the isotropic shifts of the reference molecule and the interested molecules, respectively. And the $\delta_{ref}$ is the chemical shift of the reference molecule. Here we used the Tetramethylsilane (TMS) as the standard reference molecule ($\delta_{ref}=0$). The TDDFT calculations were based on the PBE0 functional with 6-31g(d,p) basis set by the SMD model for water of Lu-CDs, and the B3LYP functional with 6-31g(d,p) basis for luteolin under an ethanol environment. We used the Multiwfn as assistance for dealing with the calculation data.

**Materials.**

The luteolin (≥98%), cis-diammineplatinum dichloride (cisplatin, 99.5%), and citric acid (≥99.5%) were purchased from Aladdin Reagent. The DSPE-mPEG (M_W_ = 5000) and DSPE-PEG-Cy5 (M_W_ = 5000) were ordered from ToYong Biotechnology. The ammonia solution (25~28%) was purchased from XiLONG Scientific. Ultrapure (UP) water was prepared by the Milli-Q-Plus system (18.2 MΩ cm^-1^) and used throughout all the experiments. The TUNEL Apoptosis Assay Kit, LDH Cytotoxicity Assay Kit, PARP Rabbit Monoclonal Antibody, and Beyo3D™ Calcein/PI Cell Viability/Cytotoxicity Assay Kit were purchased from Beyotime Biotechnology. The ROS indicator DCFH-DA was purchased from Sigma Aldrich. Annexin V-FITC Apoptosis Detection Kit was purchased from BestBio. DMEM/F-12 was purchased from Gibco (Thermo Fisher Scientific). FBS was purchased from GeminiBio. Bradford Protein Quantitation Assay Kit was purchased from Servicebio. Penicillin/streptomycin was purchased from Hyclone Laboratories, Inc.. Super ECL Detection Reagent was purchased from Yeasen Biotechnology Co., Ltd.. Immobilon®-P Membrane, PVDF, 0.45 µm was purchased from Millipore. Caspase 8/P43/P18 Monoclonal antibody, DFNA5/GSDME Monoclonal antibody, GSDMD Monoclonal antibody, and ZBP1 Polyclonal antibody were purchased from Proteintech Group, Inc.. Caspase 3 Rabbit mAb, [KD]MLKL Rabbit mAb, Phospho-MLKL (Ser345), Rabbit mAb TIM 1 Rabbit pAb, and PPAR alpha Rabbit pAb were purchased from ZEN-BIOSCIENCE. Thiazolyl Blue was purchased from MedChemExpress LLC. The immunohistochemical EliVisionTMplus detection kit was purchased from MXB Biotechnologies. Dialysis bags were ordered from Shanghai Yuanye Bio-Technology Co., Ltd. All other analytical-grade reagents were used as received without further purification.

**Synthesis of Lu-CDs.**

Briefly, 255 mg of luteolin and 3 mL of ammonia solution (25~28%) were dissolved in ultrapure water (27 mL) by sonication for 15 minutes to obtain a solution of luteolin. Subsequently, every 15 mL of the luteolin solution was added to a 25 mL Teflon-lined, stainless-steel autoclave and heated in an oven at 200 ℃ for 240 min. After the reactor was cooled to room temperature naturally, the reaction solution was filtered through 0.22 μm microporous membranes to remove the large tracts, and a transparent, dark brown solution was separated. The solution was first dialyzed in a dialysis bag with a molecular weight cut-off of 1 kDa against ultrapure water to remove residual small molecular species and ammonia. Subsequently, the above solution was further dialyzed against diluted ammonia solution (pH ≈ 9) to remove the residual small molecular species fully. Finally, the solution was dialyzed against ultrapure water again to remove the residual ammonia, and purified Lu-CDs could be obtained. A fixed volume of the solution was taken out and freeze-dried to determine the concentration of the Lu-CDs.

**Synthesis of PEGylated Lu-CDs.**

The Lu-CDs were added to the solution of DSPE-mPEG (1 mg mL^-1^) with a concentration ratio of 1:2 and then sonicated for 30 min. Then, the excess DSPE-mPEG was removed by dialysis. The solution was further filtered through 0.22 μm microporous membranes to separate a transparent dark brown solution, and PEGylated Lu-CDs were obtained. The concentrations of PEGylated Lu-CDs used in the experiments were calculated based on the content of Lu-CDs in PEGylated Lu-CDs.

**Synthesis of Cy5-labelled Lu-CDs.**

The Lu-CDs were added to the solution of DSPE-PEG-Cy5 with a concentration ratio of 1:2 and then sonicated for 30 min in the dark. Then, the excess DSPE-PEG-Cy5 was removed by dialysis. The solution was further filtered through 0.22 μm microporous membranes to separate a transparent solution, and Cy5-labelled Lu-CDs could be obtained, which were named Lu-CDs-Cy5.

**Synthesis of CDs from citric acid.**

The classical citric acid-derived CDs were prepared by a pyrolysis process of 100 g of citric acid in a muffle furnace at 220 ℃ for 48 h, according to our previous study.^[15]^ After the heating process, the product was dispersed in ultrapure water, and subsequently, the as-formed suspension was neutralized to pH 7 by the addition of NaOH aqueous solution (5 M), and the resulting suspension was filtered through 0.22 μm microporous membranes to remove the large particles. Finally, the as-prepared suspension was purified *via* dialysis in a dialysis bag with a molecular weight cut-off of 1 kDa, and the citric acid-derived CDs were obtained by freeze-drying.

**Synthesis of PEGylated CDs.**

The citric acid-derived CDs were added to the solution of DSPE-mPEG (1 mg mL^-1^) with a concentration ratio of 1:2 and then sonicated for 40 min. Then, the excess DSPE-mPEG was removed by dialysis, and PEGylated CDs could be obtained after filtering through 0.22 μm microporous membranes. The concentrations of PEGylated CDs used in the experiments were calculated based on the content of CDs in the PEGylated CDs. The CDs possessed an average diameter of ca. 3~5 nm and a ζ potential value of −27.80 ± 2.31 mV, which has been well demonstrated in our previous study. These citric acid-derived CDs are composed of graphitic carbon cores and surface oxygenated groups, including C=O, COOH, and C-OH. Moreover, PEGylated CDs were further prepared according to our previous study.^[15]^ After PEGylation, the ζ potential value of PEGylated CDs was −13.85±0.49 mV.

**Characterization.**

TEM imaging was done on the JEOL JEM-2100 transmission electron microscope. FT-IR measurements were carried out on a BRUKER Vertex 70 FT-IR spectrometer, and 32 scans were taken with a spectral resolution of 2 cm^−1^. The ζ potential measurements were performed on Malvern Nano ZS-90 at 25℃. XPS measurements were performed on a Thermo Fisher Scientific ESCALAB 250Xi XPS system. High-resolution XPS spectra were fitted using Gaussian-Lorentzian component profiles after the subtraction of a Shirley background using XPS PEAK41 software. The % L-G (the Lorentzian character in percentage) for the C 1s and O 1s was fixed at 20%. Powder X-ray diffraction (XRD) measurement was conducted on a BRUKER D8 ADVANCE X-ray diffractometer equipped with CuKα radiation (λ=0.15406 nm). UV-Vis absorbance measurements were carried out on a JASCO V-550 UV-Vis spectrophotometer. ESR analysis was conducted on a BRUKER A300 electron spin resonance spectrometer by using DMPO as the trapping agent. MALDI-TOF MS analysis was recorded on a BRUKER AutoflexIII smartbeam mass spectrometer. TGA was recorded on a Perkin-Elmer Pyris Diamond TG/DTA with a heating rate of 10 ℃/minute in the temperature range of 35 ℃~905 ℃ under a N_2_ atmosphere. Raman spectrum was recorded on a Jobin Yvon T64000 Raman spectrometer with an excitation of 532 nm.

**Solid-state NMR measurements.**

The ^13^C {^1^H} CP MAS spectra were recorded on a Bruker AVANCE NEO 400 WB spectrometer (Bruker BioSpin AG, Fällanden, Switzerland) equipped with a 4 mm standard bore CPMAS probehead whose X channel was tuned to 100.62 MHz for ^13^C and the other channel was tuned to 400.18 MHz for broad band ^1^H decoupling, using a magnetic field of 9.39T at 297 K. The dried and finely powdered samples were packed in the ZrO_2_ rotor closed with Kel-F cap which were spun at 8 kHz rate. The experiments were conducted at a contact time of 2 ms. A total of 3000 scans were recorded with 3 s recycle delay for each sample. All ^13^C CP MAS chemical shifts are referenced to the resonances of adamantane (C_10_H_16_) standard (d_CH2_=38.4).

**Electron spin resonance spectroscopy.**

Electron spin resonance (ESR) spectroscopy was adopted to explore the radical scavenging activity of Lu-CDs towards OH· than ·O_2_^-^ by using DMPO as the trapping agent (33 mM). The characteristic peak signals were recorded on an ESR spectrometer. In these experiments, the intensities of the ESR signal were measured as the peak-to-peak height of the second line of the ESR spectrum. The ESR settings: microwave power: 19.23 mW, microwave frequency: 9.853616 GHz, center field: 3510.00 G, modulation frequency: 100.00 kHz; modulation amplitude: 1.00 G.

**Cell culture.**

HK-2 cells and LX-2 cells were obtained from the American Type Culture Collection (ATCC) and cultured at 37 ℃ under 5% CO_2_ in an incubator. Media was Dulbecco’s Modified Eagle Medium/Nutrient Mixture F-12 (DMEM/F12) containing FBS (10%), and penicillin/streptomycin (1%, W/V) for HK-2 cells, Dulbecco’s Modified Eagle Medium (DMEM, high glucose) containing FBS (10%), and penicillin/streptomycin (1%, W/V) for LX-2 cells. For in *vitro* studies, the concentration of the Lu-CDs or CDs used was 25 μg/mL, while the luteolin was applied at its optimal concentration of 5 μM. Higher concentrations of luteolin induced significant cytotoxicity, whereas lower concentrations of luteolin elicited insufficient therapeutic efficacy. For direct comparison, the samples including Lu-CDs and reference PEGylated CDs were tested at the same concentration *in vitro.*

**Cell viability study.**

To investigate the detoxification activities of PEGylated Lu-CDs against cisplatin-induced cytotoxicity, HK-2 cells (1×10^4^ cells per well) were seeded in 96-well plates and incubated overnight. After incubating with cisplatin (15 μg/mL) together with different concentrations of PEGylated Lu-CDs for 24 h, MTT was added to the culture medium and incubated for 4 h at 37 ℃ in 5% CO_2_.^[15]^ Then, 200 μL of DMSO was added to dissolve the formazan crystals, and a microplate reader (BMG LABTECH CLARIOstar®Plus) was used to measure the absorbance at 570 nm.

***In vitro* live/dead cells staining assay.**

HK-2 cells (12 × 10^4^ cells per well) were plated in 6-well plates and cultured overnight. Subsequently, they were exposed to different formulations for 24 h before being incubated with Calcein/PI for 30 min in the dark at 37 ℃. Finally, they were observed under an Olympus fluorescence microscope and photographed.

**Lactic dehydrogenase detection.**

The HK-2 cells (1.2 × 10^5^ cells per well) were stimulated with different formulations for 24 h at 37 ℃. The lactic dehydrogenase (LDH) levels in the supernatant were examined by the LDH Cytotoxicity Assay Kit according to the manufacturer’s protocols.

**Intracellular ROS detection.**

Intracellular ROS level was detected by flow cytometry with DCFH-DA. Briefly, HK-2 cells (5×10^5^ cells per well) were seeded in 6-well plates and incubated overnight. After incubating with cisplatin (15 μg/mL) together with PEGylated Lu-CDs for 24 h, cells were stained with DCFH-DA for 20 min at 37 ℃ in 5% CO_2_. Then, the cells were collected for fluorescence intensity detection by flow cytometry (BD LSRFortessa Cell Analyzer).

**Apoptosis assay.**

Cell apoptosis assays were performed using the Annexin V-FITC Apoptosis Detection Kit following the manufacturer’s instructions. Briefly, HK-2 cells (5×10^5^ cells per well) were seeded in 6-well plates and incubated overnight. After incubating with cisplatin (15 μg/mL) together with PEGylated Lu-CDs for 24 h, cells were stained with Annexin V-FITC and Propidium Iodide (PI) for 15 min at 37 ℃ in 5% CO_2_, and subsequently collected and analyzed by flow cytometry (BD LSRFortessa Cell Analyzer).

**Western blotting.**

Samples were first lysed in RIPA buffer supplemented with the protease inhibitor cocktail at 4 ℃, and the protein concentration was determined by a Quick Start Bradford protein assay Kit. Afterwards, 25 μg of protein from each sample was resolved by SDS-PAGE (8%-10% SDS-PAGE gels), and subsequently transferred to the polyvinylidene difluoride (PVDF) membranes. Membranes were then blocked in 5% skim milk in TBST and probed with specific primary antibodies. HRP-conjugated secondary antibodies and ECL Chemiluminescence Detection Kit were used for protein detection. The level of β-actin immunoreactivity was used as a control to monitor equal protein loading**.**

**Animals.**

Female Balb/c mice (6-8 weeks) and female Kunming mice (6-8 weeks) were purchased from Charles River Laboratories. In this study, the animal studies were approved by the ethics committee of Beijing Institute of Pharmacology and Toxicology (Approval number: IACUC-DWZX-2025-P565). The female Balb/c mice were used to investigate the biosafety of PEGylated Lu-CDs as well as the therapeutic efficacy of PEGylated Lu-CDs for AKI therapy, whereas the female Kunming mice were used to perform the hemolysis test of PEGylated Lu-CDs.

**Hemolysis test of PEGylated Lu-CDs.**

400 μL of the whole blood was collected in tubes containing EDTA-K2 from the orbital venous of mice. Then, blood samples were mixed with the proper amount of PBS (10 mM, pH = 7.4), centrifuged for 5 min at 3000 rpm, discarded the supernatant, and repeated 3~4 times until the supernatant became colorless and transparent. The precipitated erythrocytes were dispersed in 4 mL PBS (10 mM, pH = 7.4) to get erythrocyte suspensions. The PEGylated Lu-CDs and erythrocyte suspensions were added to the PBS (10 mM, pH = 7.4) respectively to obtain a final PEGylated Lu-CDs concentration as 6.25 μg mL^-1^, 12.5 μg mL^-1^, 25 μg mL^-1^, and 50 μg mL^-1^, and only erythrocyte suspension was added in 10 mM PBS (pH = 7.4) to get the negative control, and the positive control was erythrocyte suspension diluted with ultrapure water. The tubes were incubated for 8 h at 4 ℃, and the hemolysis phenomenon was observed and recorded. Meanwhile, the above mixtures were centrifuged, and the absorbance of the supernatants at 540 nm was determined by a microplate reader (BMG LABTECH CLARIOstar®Plus). Calculation of the hemolysis rate (HR%): HR% = (A_Lu-CDs_-A_NC_) * 100%/(A_PC_-A_NC_), where A_Lu-CDs_, A_PC_, and A_NC_ were the absorbance of the sample, the positive control, and the negative control, respectively.

***In vivo* biosafety of PEGylated Lu-CDs.**

Balb/c mice were randomly divided into two groups. The mice without any treatments were defined as the control group, and the mice with intravenous injection of PEGylated Lu-CDs (4 mg Lu-CDs/kg) were defined as the test group. The body weight of mice was measured every three days to evaluate the *in vivo* bio-safety with a total experimental period of 21 days. At the indicated time points, including 1 day, 7 days, and 21 days, the blood of mice in all groups was collected, and the samples were used to perform blood biochemistry and hematology analysis. Moreover, the major organs, including the heart, liver, spleen, lung, and kidney, were harvested, fixed in 4% paraformaldehyde, processed into paraffin, sectioned, and stained with hematoxylin and eosin (H&E).

**Cisplatin-induced AKI mouse model.**

All female Balb/c mice (6-8 weeks) were adapted for 7 days before being administered an intraperitoneal injection of cisplatin (15 mg/kg).^[15]^ The time point at 24 h post-injection of cisplatin was defined as the initiation of AKI.

Regarding the selection of the mouse sex for the studies of cisplatin-induced nephrotoxicity, some studies have reported that female sex is resistant than male sex due to greater antioxidant defense and protective effects of estrogen in females. Yet, other studies have indicated that males are less vulnerable than females due to cisplatin’s high clearance. Thus, whether sex confers susceptibility or resistance to cisplatin nephrotoxicity remains an area of significant controversy.^[16]^ Hence, the selection of female mice for this preliminary efficacy study was based on practical considerations for model stability and animal welfare. First, it avoids the hierarchy-related aggression common in group-housed males, which induces stress and physiological variability that could confound renal function assessment. Female groups provide a more stable baseline for a preliminary efficacy study. Second, their smaller size and calmer nature facilitate safer and less stressful handling during procedures like injections and imaging. We recognize the critical role of sex differences. This work establishes a proof-of-concept in females, and based on these promising findings, a systematic comparison including male mice is a defined next step in the future research plan.

**Biodistribution analysis.**

For biodistribution analysis, mice were intravenously injected with Lu-CDs-Cy5 to carry out fluorescence imaging. At different expected time points, mice were sacrificed, and major organs were collected and imaged. Images were analyzed *via* ImageJ Software.

**AKI therapy *in vivo* by PEGylated Lu-CDs.**

At the time point of the initiation of AKI, different treatments were performed: group 1 was the control healthy mice (n = 5); group 2 was the control AKI mice (n = 5); group 3 was the AKI mice treated with luteolin (4 mg/kg, n = 5); group 4 was the AKI mice treated with PEGylated CDs (4 mg CDs/kg, n = 5); group 5 was the AKI mice treated with PEGylated Lu-CDs (4 mg Lu-CDs/kg, n = 10). On day 3 after various treatments, mice in the groups of 1, 2, 3, 4, and five mice in group 5 were euthanized, and the remaining five mice in group 5 were euthanized on day 7. Afterwards, the renal functions of mice in different groups were explored. Moreover, the kidneys of mice in various experimental groups were collected for histological analysis and immunohistochemical staining. For direct comparison, all samples including luteolin, Lu-CDs, and PEGylated CDs were tested at the same concentration *in vivo.*

**Bodyweight analysis.**

Bodyweight variations of the AKI mice were monitored for 7 days after different treatments. According to the guidelines on animal welfare, a bodyweight loss exceeding 20% in mice was considered a humane endpoint, necessitating euthanasia.

**Renal function analysis.**

Renal function tests were performed to investigate the therapeutic efficacy of PEGylated Lu-CDs for AKI treatment. After 3 days or 7 days post-injection, mice were sacrificed to collect the blood samples for the detection of the serum levels of urea nitrogen (BUN) and serum creatinine (CREA).

**Histological analysis.**

At different indicated time points, mice were sacrificed, and the kidneys were collected for histological analysis. Harvested kidneys were fixed with paraformaldehyde (4%), dehydrated, embedded in paraffin, and sectioned for hematoxylin and eosin (H&E) staining and TUNEL staining.

**Statistical analysis.**

All data were expressed as mean ± standard deviation (SD) and were obtained from at least 3 specimens. The GraphPad 6.0 software was used to analyze experimental data. The Mann-Whitney test or one-way analysis of variance (Dunnett’s t-test) was performed on the studied data. A *P* value <0.05 was considered statistically significant. Asterisks indicated significant differences (**P* < 0.05, ***P* < 0.01, ****P* < 0.001, *****P* < 0.0001).

**II. Figures**


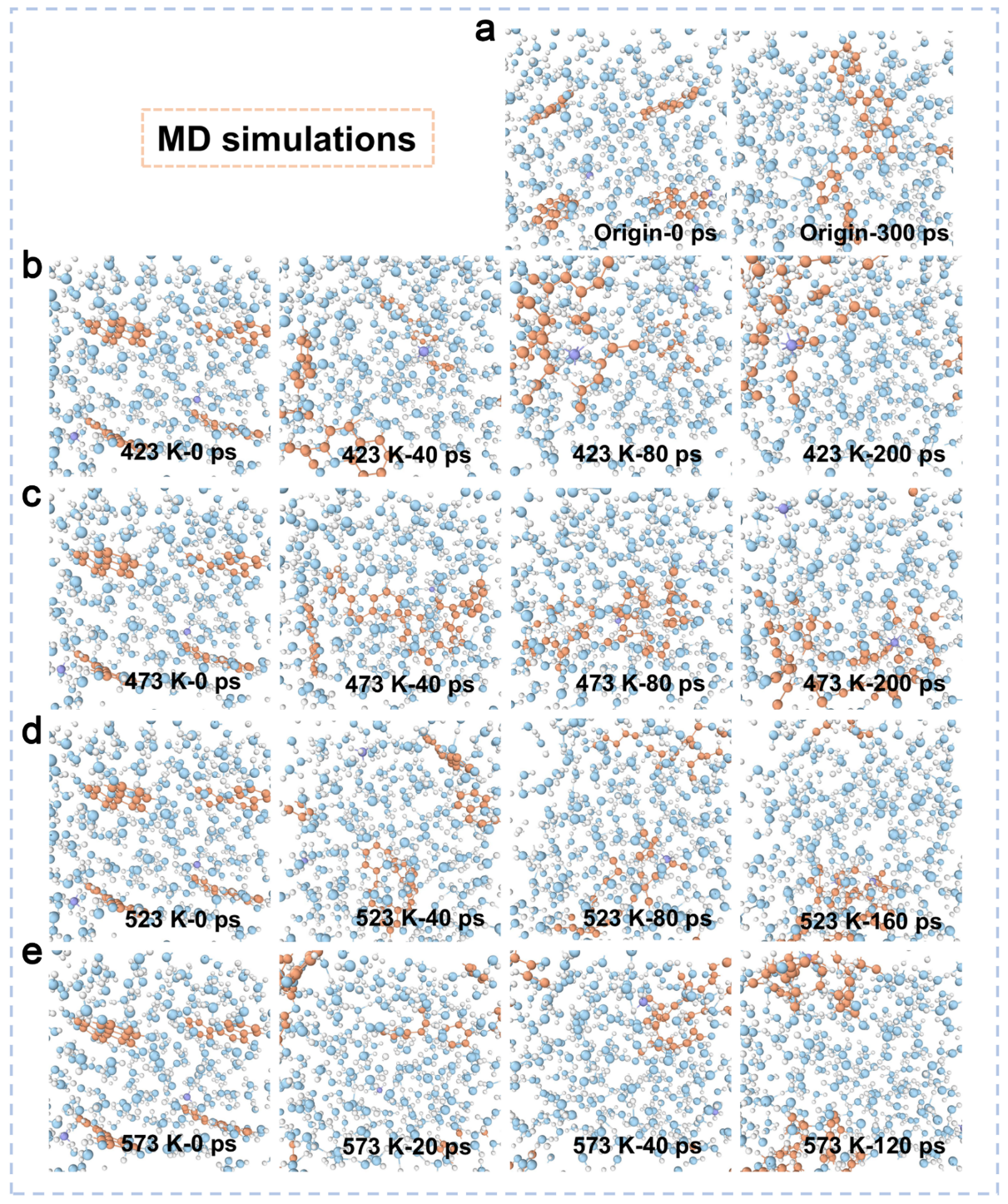


**Figure S1.** The simulated processes of luteolin under different temperatures. (a) The classical MD simulations within 300 ps. (b) MetaD at 423 K. (c) MetaD at 473 K. (d) MetaD at 523 K. (e) MetaD at 573 K.


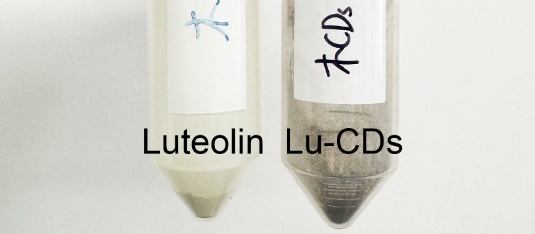


**Figure S2.** Typical digital photograph of luteolin (left) and Lu-CDs (right) powders.


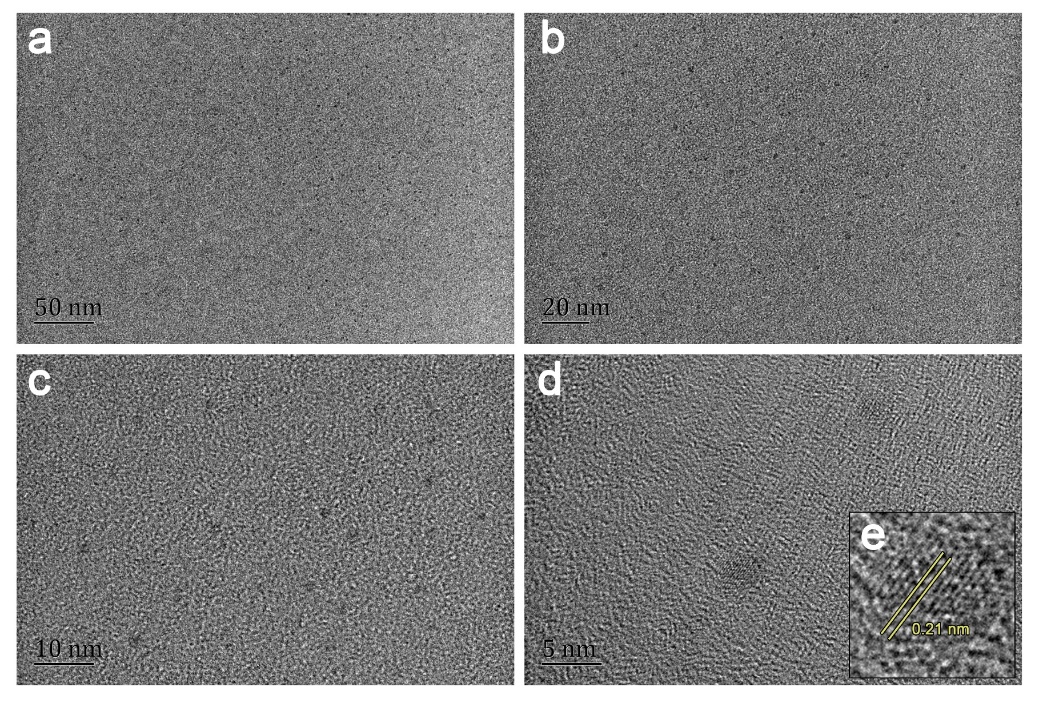


**Figure S3.** Typical TEM and HR-TEM images of Lu-CDs at different magnifications. Inset: Lu-CDs hold fine crystallinity with a lattice spacing of 0.21 nm.





**Figure S4.** Size distributions of Lu-CDs as determined *via* Nano Measurer software by analyzing 100 dots. The average size of Lu-CDs is 1.95 nm. The maximum size and the minimum size of Lu-CDs are 3.04 nm and 1.13 nm, respectively.





**Figure S5.** The DTG profiles of luteolin and Lu-CDs.


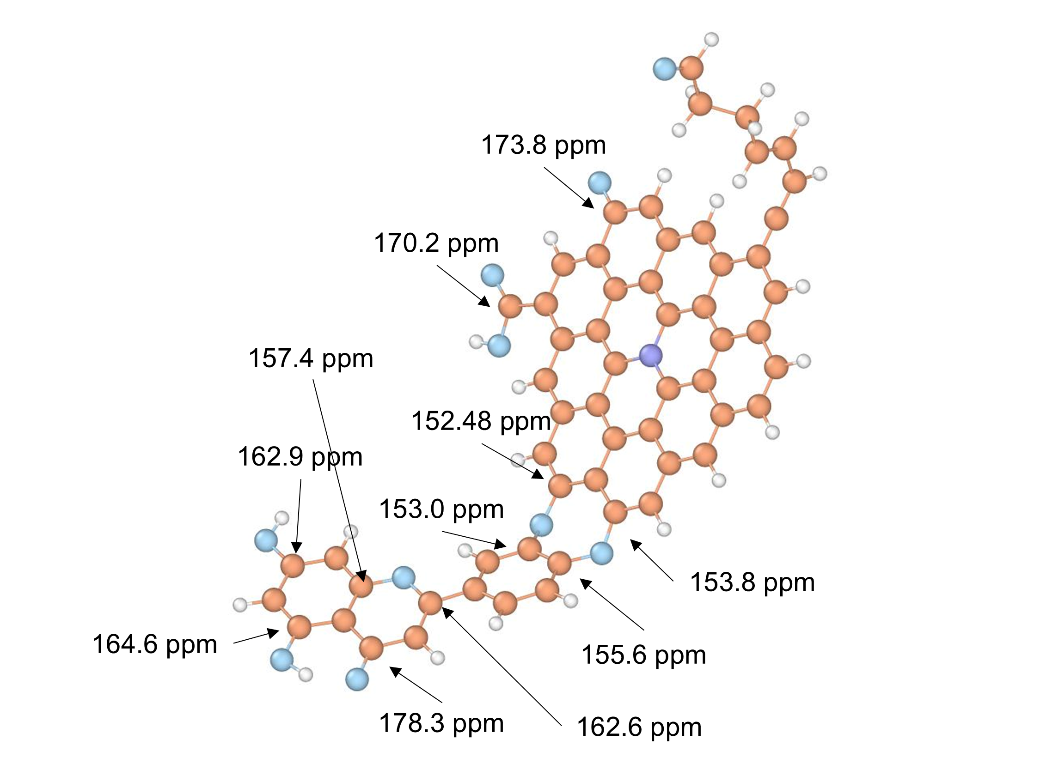


**Figure S6.** Some calculated chemical shifts of Lu-CDs-1. The calculated values are, on average, +6.5 ppm larger than the actual system values.


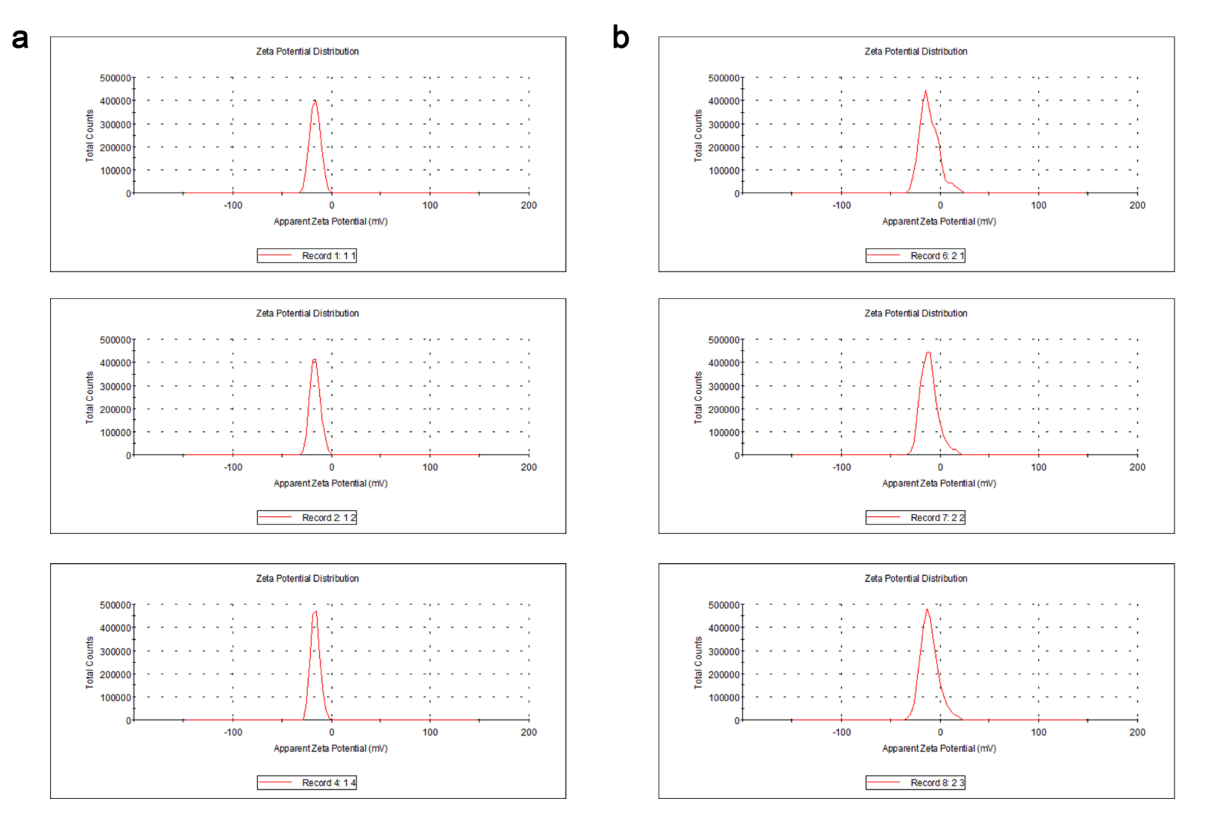


**Figure S7.** Zeta potential distributions of Lu-CDs (a) and PEGylated Lu-CDs (b).


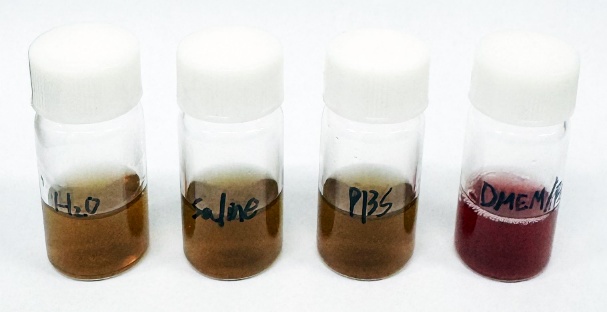


**Figure S8.** Photograph of Lu-CDs without PEGylation dispersed in water, saline, PBS, and DMEM (10% FBS). Lu-CDs without PEGylation show poor biostability with flocculation in the above media after being placed for 1 h.


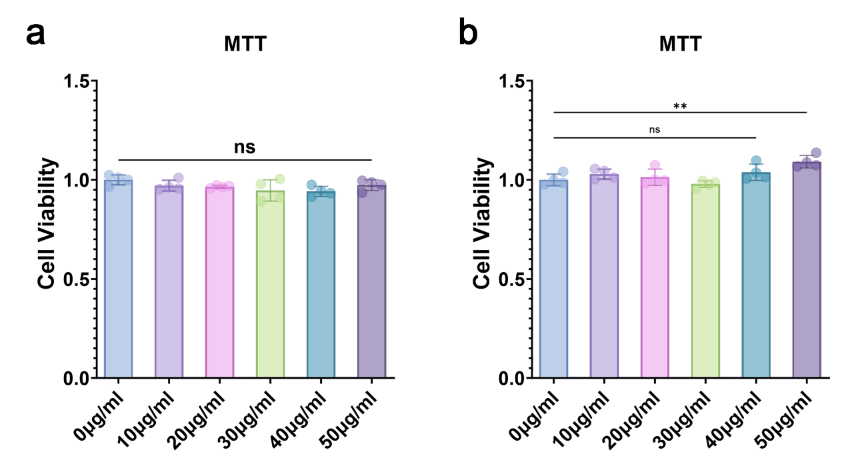


**Figure S9.** Cytotoxicity of Lu-CDs towards (a) HK-2 cells and (b) LX-2 cells. Data represent mean ± standard deviation (n = 4). The Mann-Whitney test or one-way analysis of variance (Dunnett’s t-test) was performed on the studied data. Asterisks indicate statistically significant differences (**P* < 0.05, ***P* < 0.01, ****P* < 0.001, and *****P* < 0.0001).


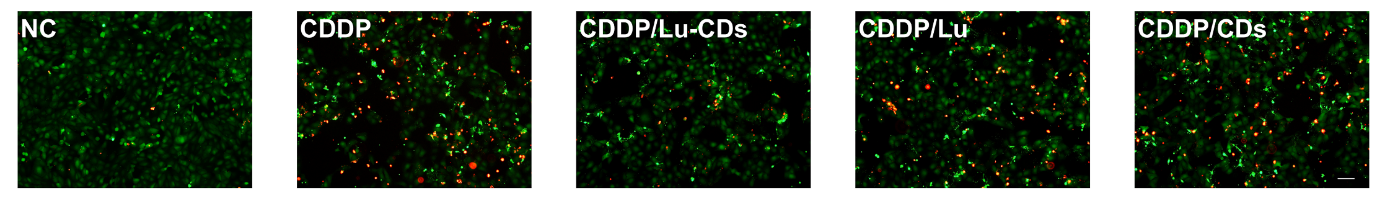


**Figure S10.** Live/dead staining fluorescence images of HK-2 cells in various experimental groups. The scale bar is equal to 100 μm.


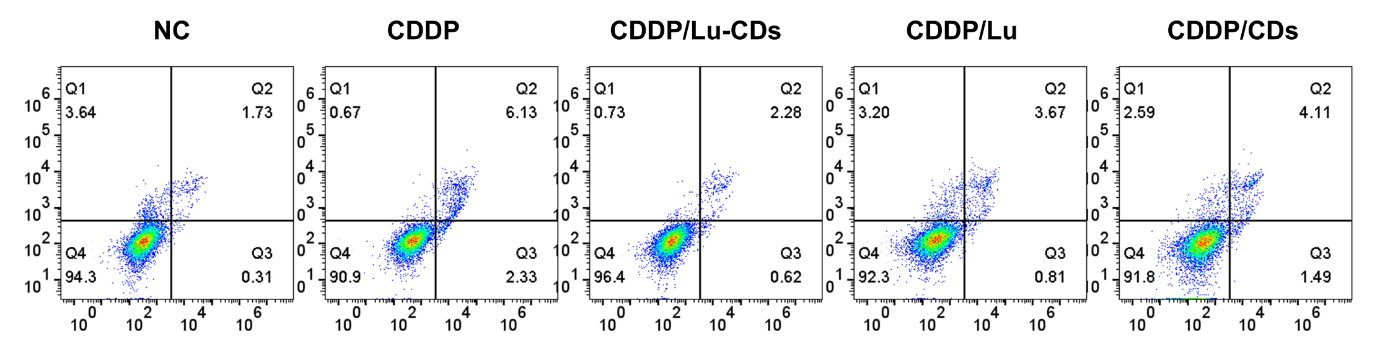


**Figure S11.** Apoptosis analysis of HK-2 cells after various treatments based on flow cytometry.


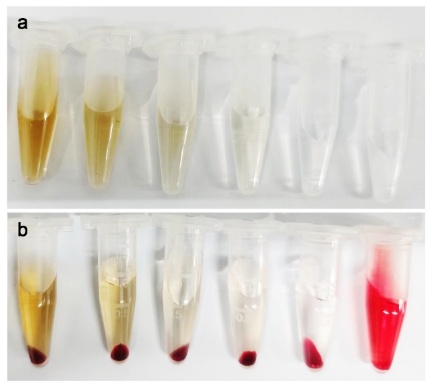


**Figure S12.** Typical digital photographs of concentration-dependent hemolysis of PEGylated Lu-CDs. (a) PEGylated Lu-CDs with various concentrations. From left to right: photographs of PEGylated Lu-CDs under concentrations of 50 μg Lu-CDs mL^-1^, 25 μg Lu-CDs mL^-1^, 12.5 μg Lu-CDs mL^-1^, 6.25 μg Lu-CDs mL^-1^, PBS, and ultrapure water. (b) Concentration-dependent hemolysis of PEGylated Lu-CDs. From left to right: hemolysis of PEGylated Lu-CDs under concentrations of 50 μg Lu-CDs mL^-1^, 25 μg Lu-CDs mL^-1^, 12.5 μg Lu-CDs mL^-1^, 6.25 μg Lu-CDs mL^-1^, PBS (the negative control), and ultrapure water (the positive control).


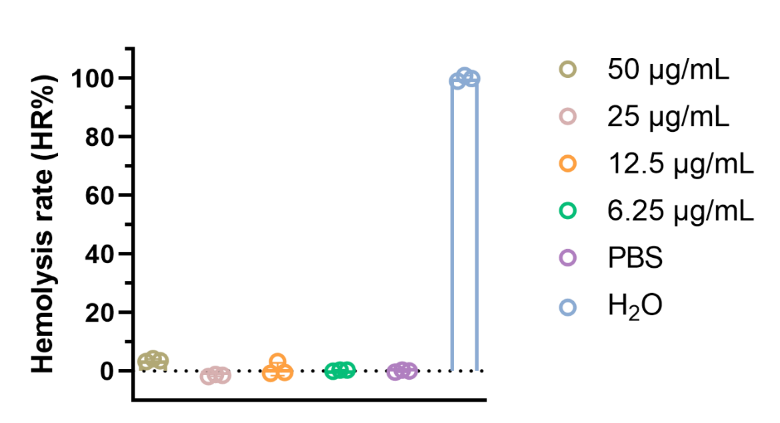


**Figure S13.** Results of hemolytic assay. Concentration-dependent hemolysis of PEGylated Lu-CDs. PBS and ultrapure water were regarded as the negative and positive controls, respectively. Error bars represent the standard deviation from the mean (n = 3).


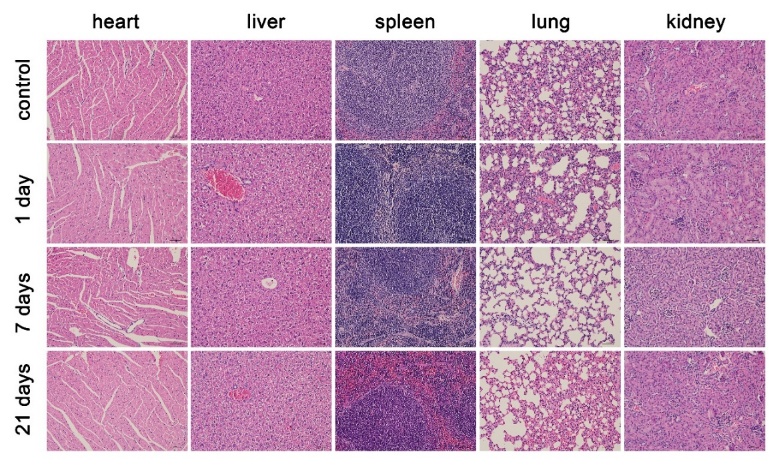


**Figure S14.** H&E-stained images of the slices of the major organs collected from mice after intravenous injection of PEGylated Lu-CDs at the expected time points. The mice were intravenously injected with PEGylated Lu-CDs (4 mg Lu-CDs kg^-1^) for various experimental groups. No significant tissue abnormalities are observed in the H&E-stained images. The scale bars are equal to 50 μm.


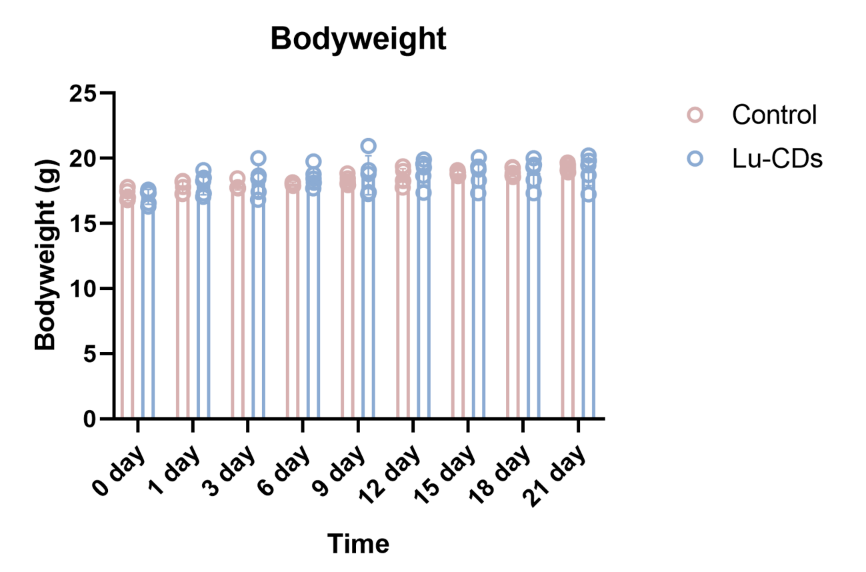


**Figure S15.** Time-dependent bodyweight variations of healthy mice after intravenous injection of PEGylated Lu-CDs. Error bars represent the standard deviation from the mean (n = 5).


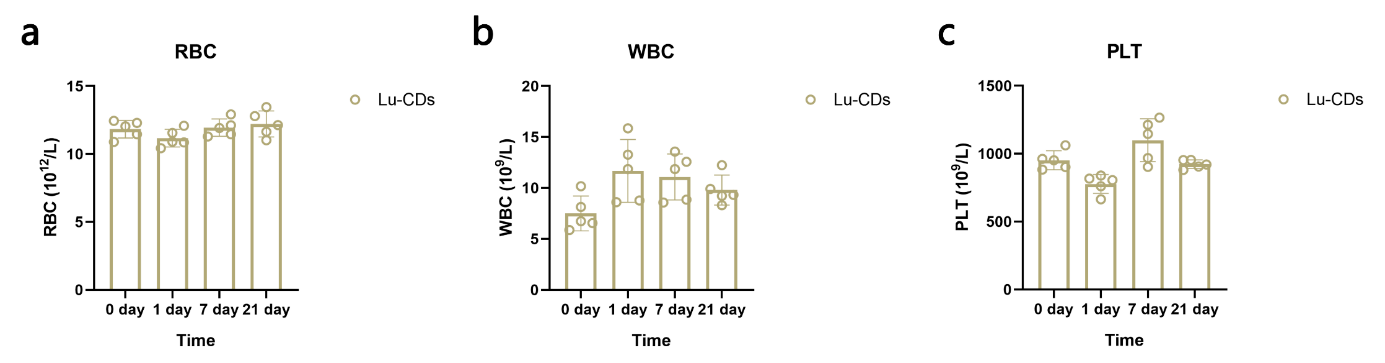


**Figure S16.** Results of hematological analysis. (a) RBC, red blood cells. (b) WBC, white blood cells. (c) PLT, platelets. Error bars represent the standard deviation from the mean (n = 5).


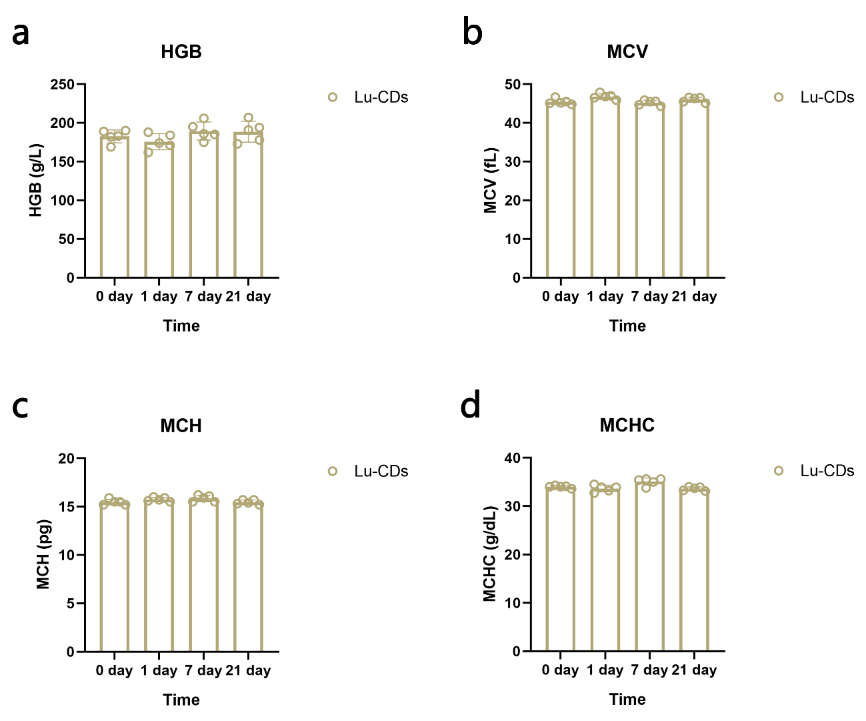


**Figure S17.** Results of hematological analysis. (a) HGB, hemoglobin. (b) MCV, mean corpuscular volume. (c) MCH, mean corpuscular hemoglobin. (d) MCHC, mean corpuscular hemoglobin concentration. Error bars represent the standard deviation from the mean (n = 5).


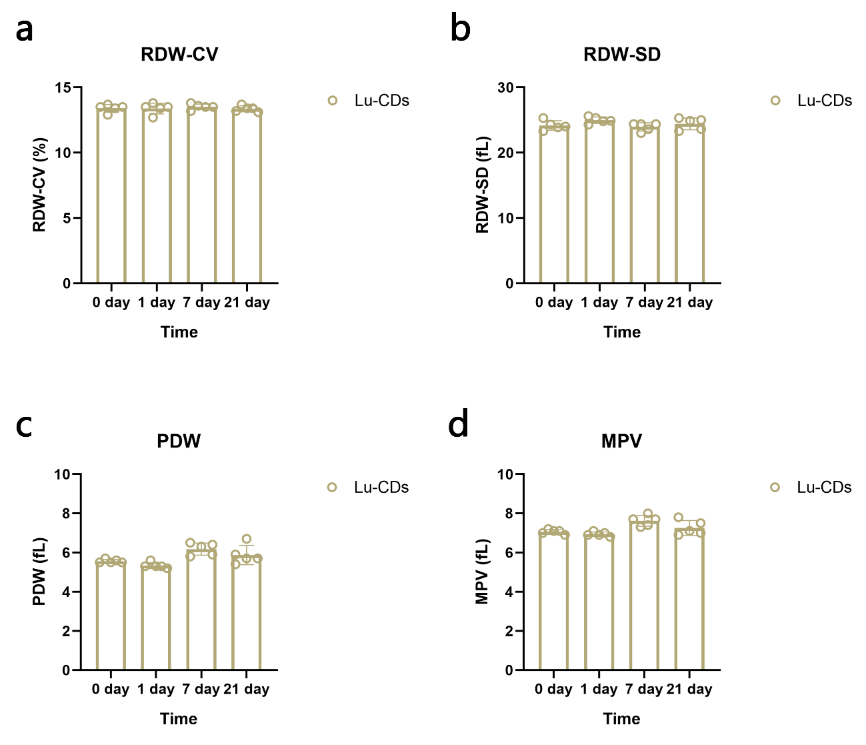


**Figure S18.** Results of hematological analysis. (a) RDW-CV, red blood cell distribution width-coefficient of variation. (b) RDW-SD, red blood cell distribution width-standard deviation. (c) PDW, platelet distribution width. (d) MPV, mean platelet volume. Error bars represent the standard deviation from the mean (n = 5).


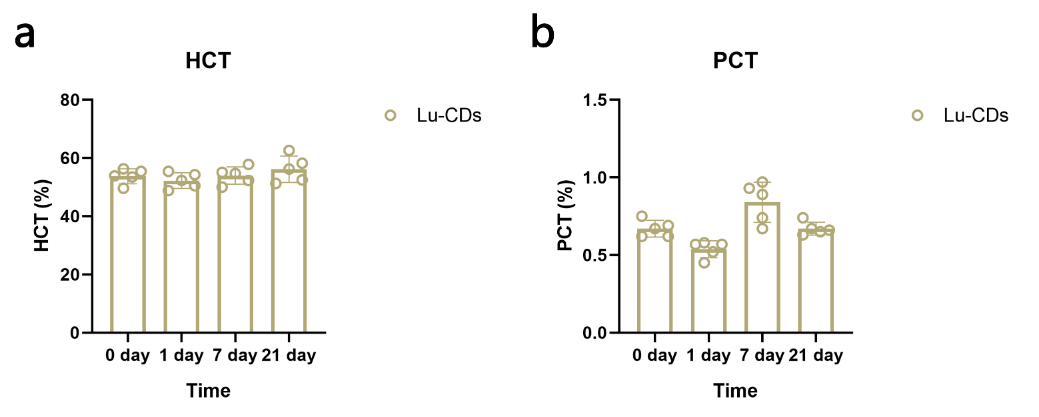


**Figure S19.** Results of hematological analysis. (a) HCT, hematocrit. (b) PCT, plateletcrit. Error bars represent the standard deviation from the mean (n = 5).


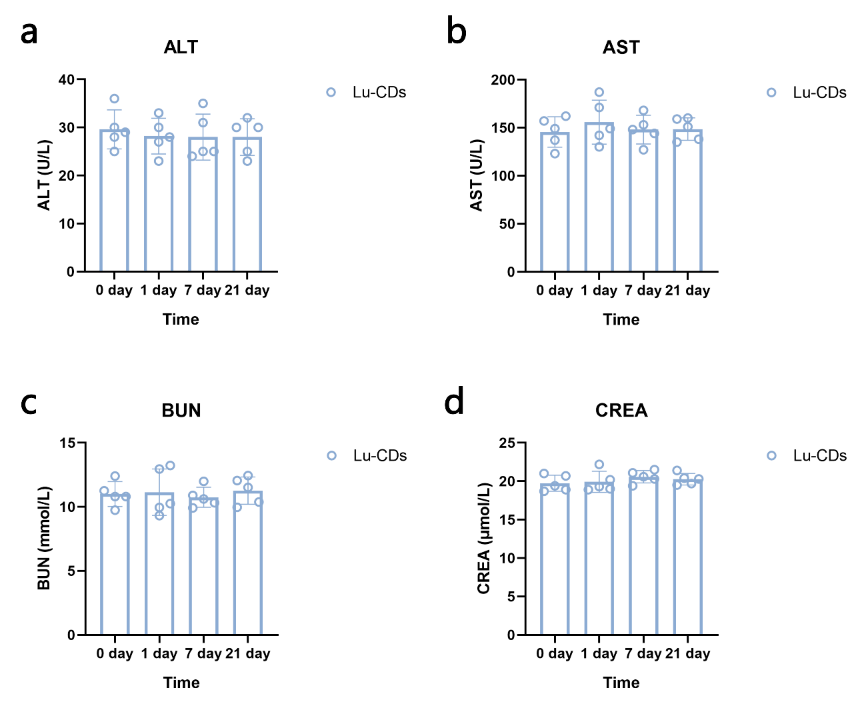


**Figure S20.** Results of blood biochemistry assay. (a) ALT, alanine aminotransferase. (b) AST, aspartate aminotransferase. (c) BUN, blood urea nitrogen. (d) CREA, serum creatinine. Error bars represent the standard deviation from the mean (n = 5).


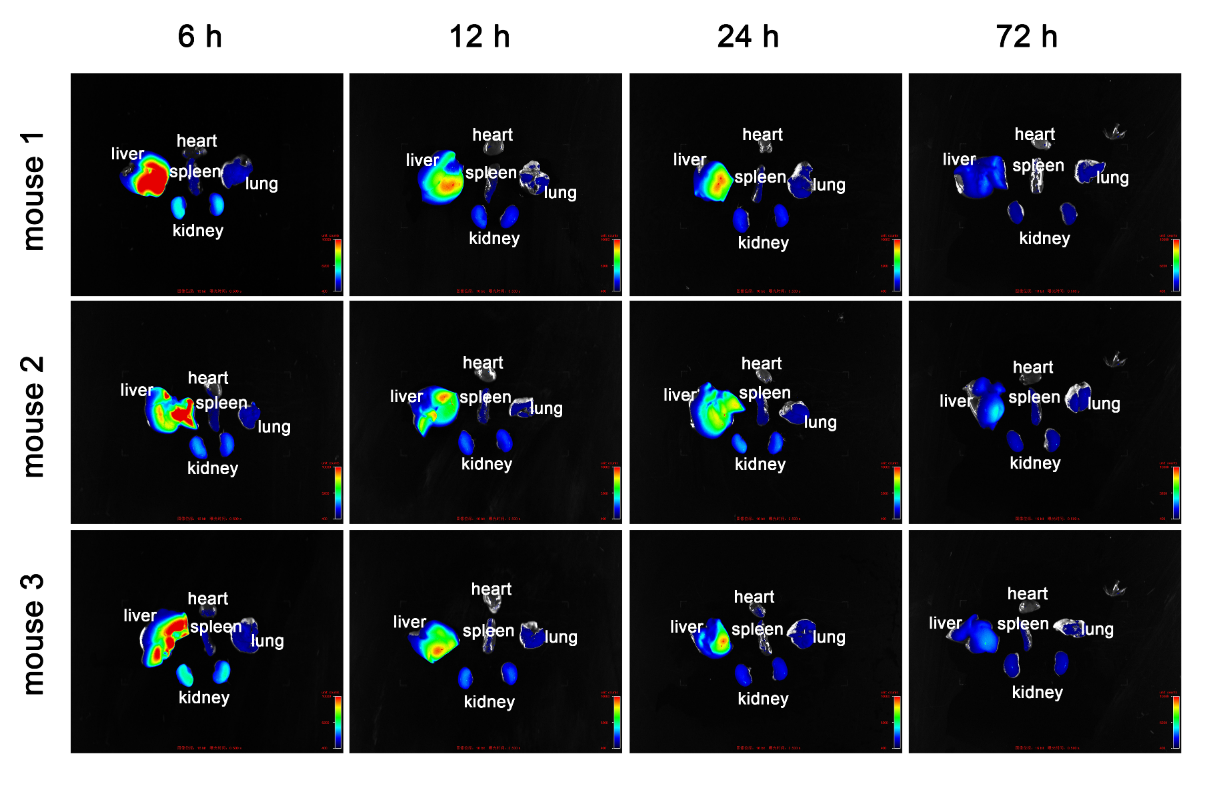


**Figure S21.** Time-dependent *ex vivo* fluorescence imaging of mice after intravenous injection of Lu-CDs-Cy5.


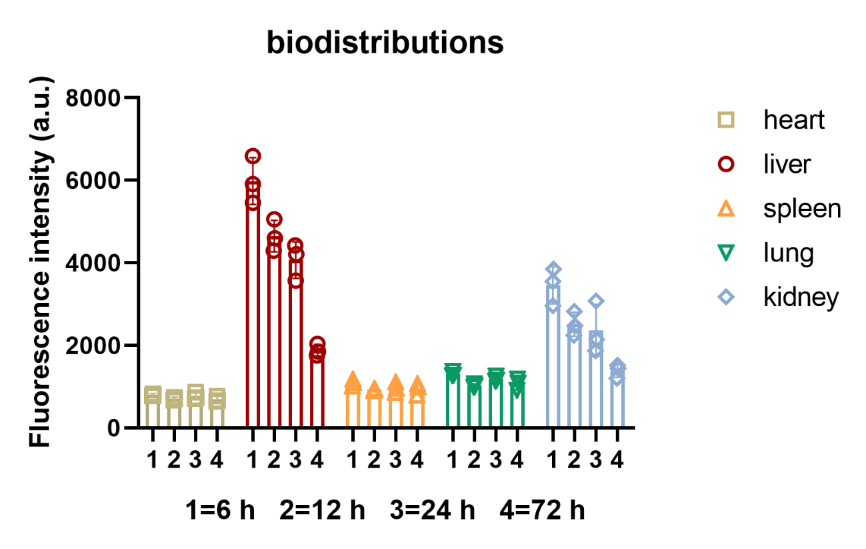


**Figure S22.** Time-dependent biodistributions of Lu-CDs-Cy5 quantified by the *ex vivo* fluorescence images using ImageJ software. Error bars represent the standard deviation from the mean (n = 3).


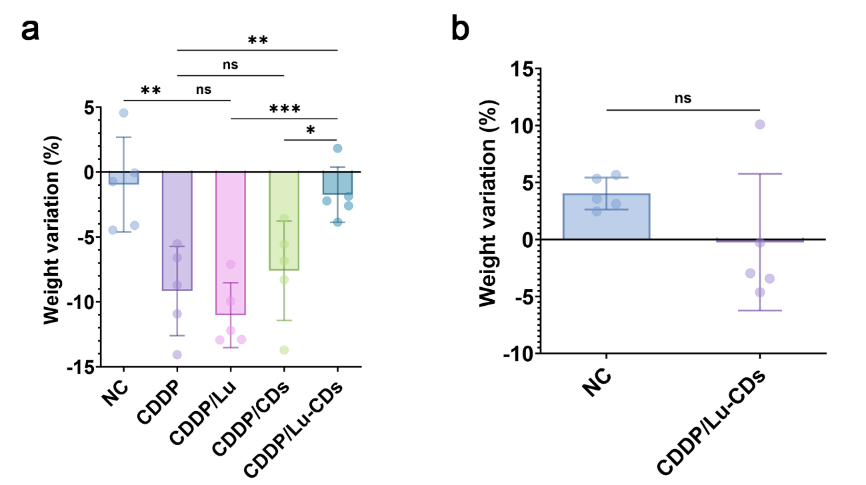


**Figure S23.** Bodyweight changes of AKI mice after various treatments on day 2 (a) and day 7 (b) post-AKI induction. Data represent mean ± standard deviation (n = 5). The Mann-Whitney test or one-way analysis of variance (Dunnett’s t-test) was performed on the studied data. Asterisks indicate statistically significant differences (**P* < 0.05, ***P* < 0.01, ****P* < 0.001, and *****P* < 0.0001).


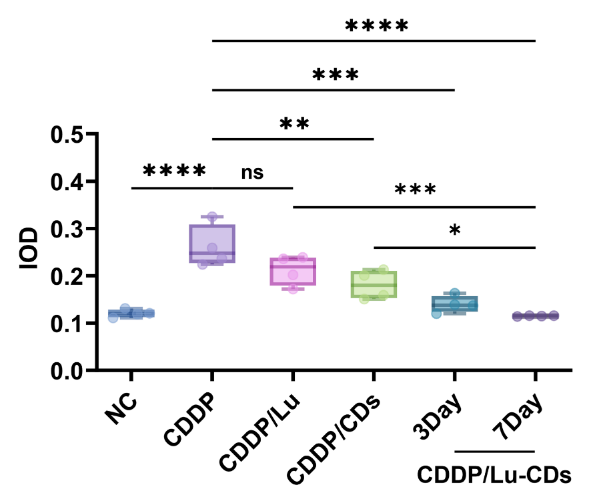


**Figure S24.** Quantitative analysis of TUNEL staining images. Data represent mean ± standard deviation (n = 4). The Mann-Whitney test or one-way analysis of variance (Dunnett’s t-test) was performed on the studied data. Asterisks indicate statistically significant differences (**P* < 0.05, ***P* < 0.01, ****P* < 0.001, and *****P* < 0.0001).


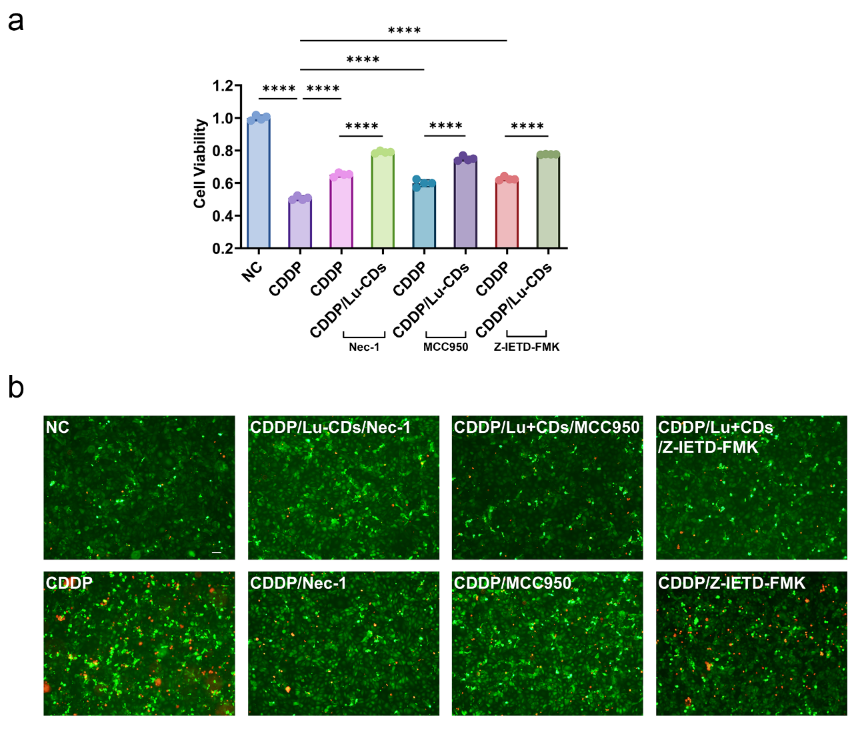


**Figure S25.** (a) Cell viability of HK-2 cells in various experimental groups. (b) Live/dead staining fluorescence images of HK-2 cells in various experimental groups. The scale bar is equal to 100 μm. The Lu-CDs or CDs used were at 25 μg mL^-1^, while luteolin was applied at its optimal concentration of 5 μM. The pharmacological inhibitors used were 25 μM. Data represent mean ± standard deviation (n = 4). The Mann-Whitney test or one-way analysis of variance (Dunnett’s t-test) was performed on the studied data. Asterisks indicate statistically significant differences (**P* < 0.05, ***P* < 0.01, ****P* < 0.001, and *****P* < 0.0001).


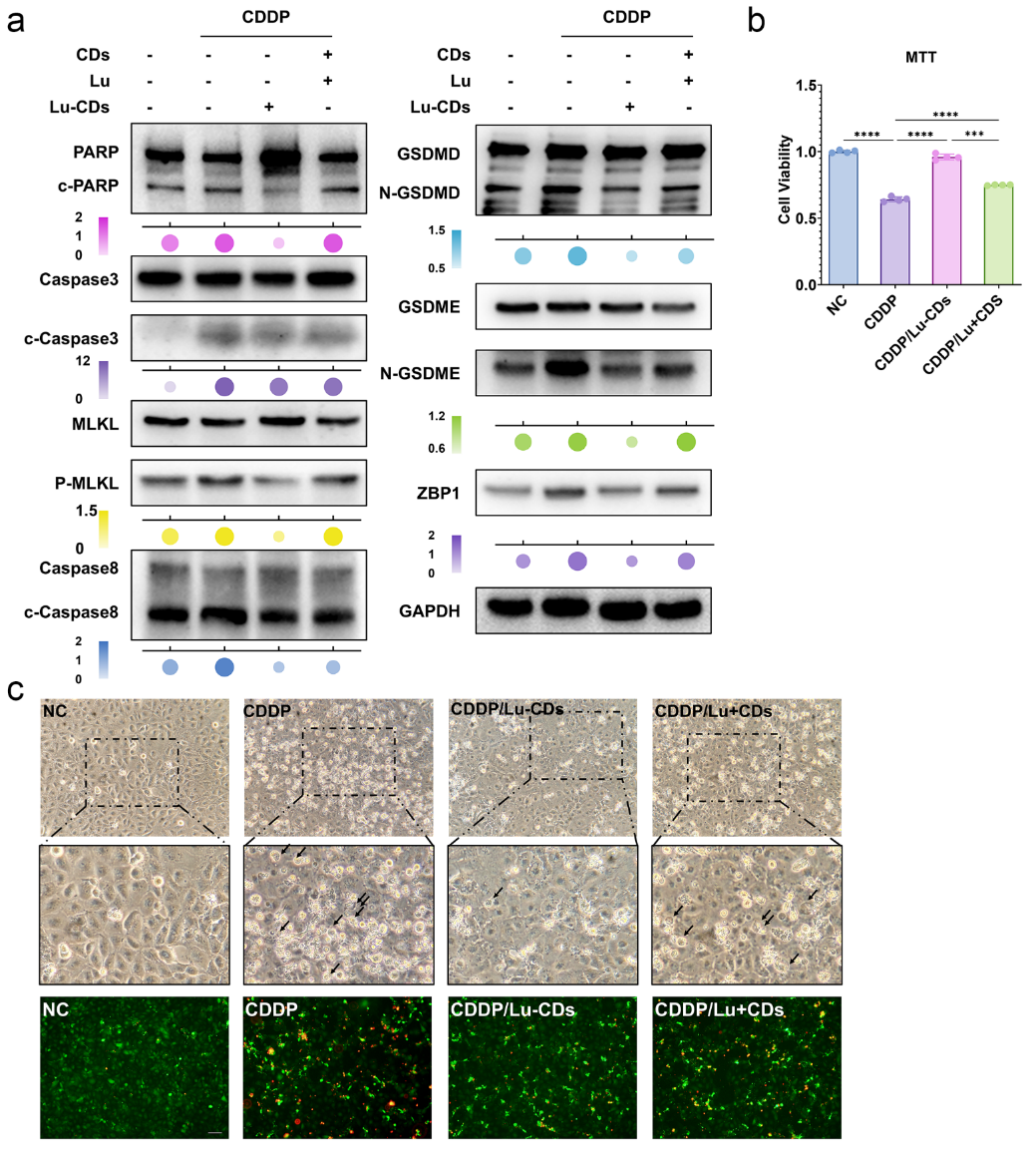


**Figure S26.** (a) Western blot analysis of the biomarkers for PANoptosis in various experimental groups. The color intensity and size of the colored bubbles correspond to relative protein expression levels, with darker hues and larger diameters indicating higher expression. (b) Cell viability of HK-2 cells in various experimental groups. (c) Typical bright-field images and Live/dead staining fluorescence images of HK-2 cells in various experimental groups. The scale bar is equal to 100 μm. Lu+CDs indicate the physical mixture of CDs and free luteolin. The Lu-CDs or CDs used were at 25 μg mL^-1^, while luteolin was applied at its optimal concentration of 5 μM. Data represent mean ± standard deviation (n = 4). The Mann-Whitney test or one-way analysis of variance (Dunnett’s t-test) was performed on the studied data. Asterisks indicate statistically significant differences (**P* < 0.05, ***P* < 0.01, ****P* < 0.001, and *****P* < 0.0001).


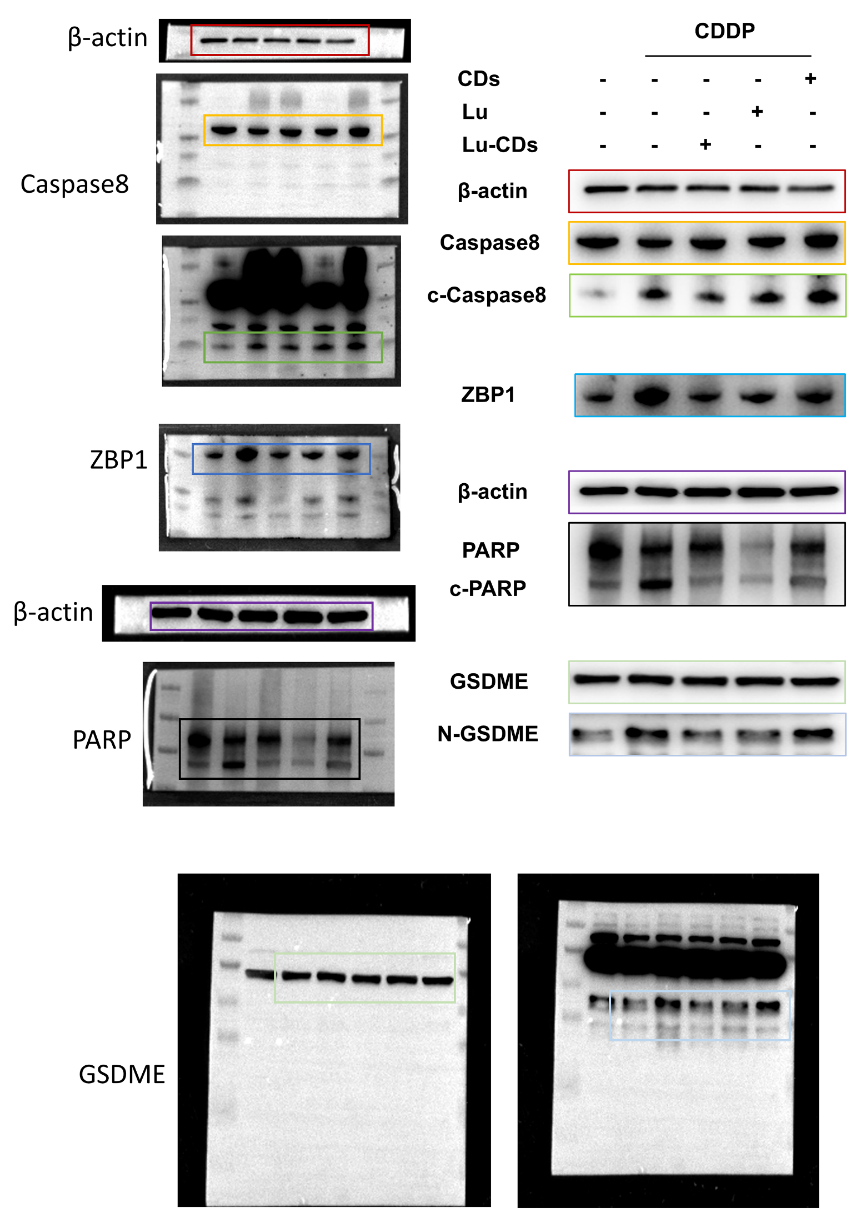


**Figure S27.** Related to **Figure 5e**. The uncropped western blot images of the western blot analysis of the expression of PANoptosis biomarkers in HK-2 cells in various experimental groups. The immunoblot bands in the colored box of the uncropped western blot images are the raw data of the processed western blot data marked by the box of the same color.


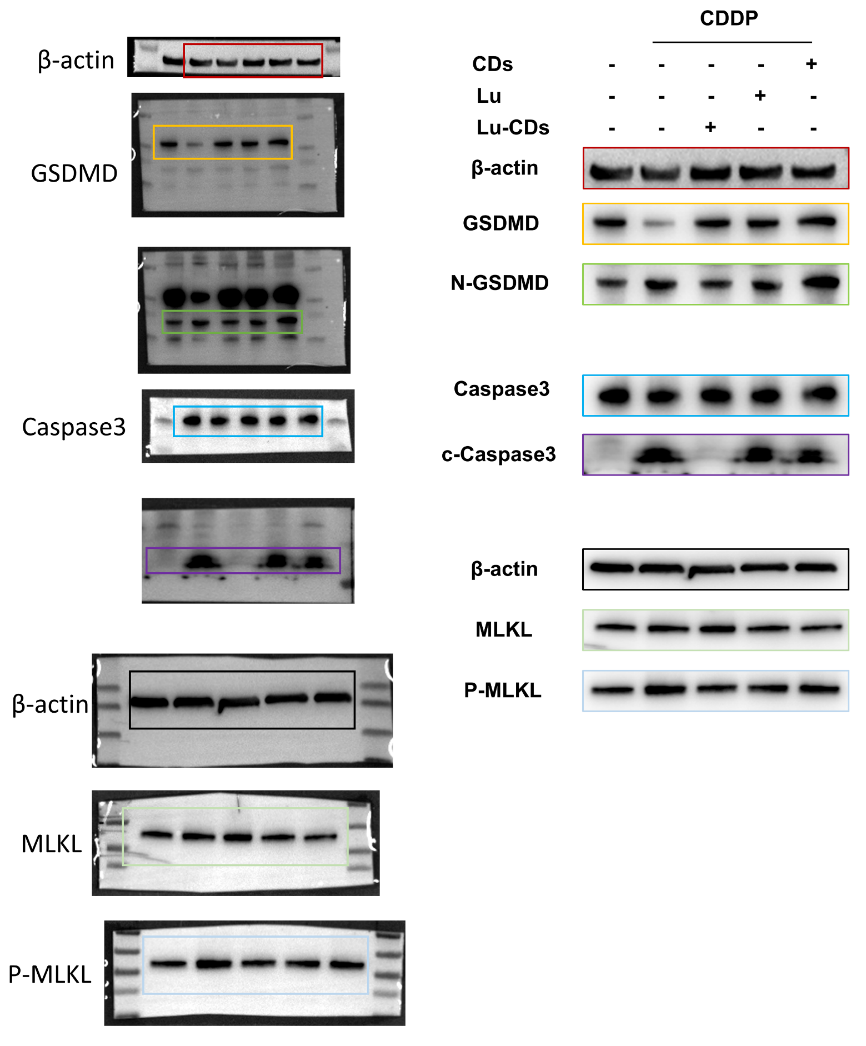


**Figure S28.** Related to **Figure 5e**. The uncropped western blot images of the western blot analysis of the expression of PANoptosis biomarkers in HK-2 cells in various experimental groups. The immunoblot bands in the colored box of the uncropped western blot images are the raw data of the processed western blot data marked by the box of the same color.


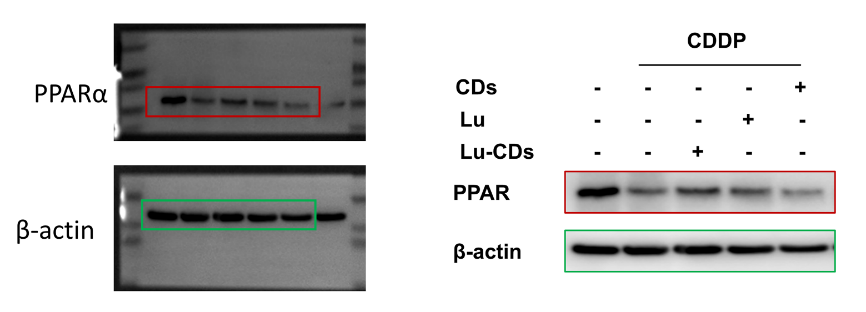


**Figure S29.** Related to **Figure 7a**. The uncropped western blot images of the western blot analysis of the expression of PPARα in HK-2 cells in various experimental groups. The immunoblot bands in the colored box of the uncropped western blot images are the raw data of the processed western blot data marked by the box of the same color.


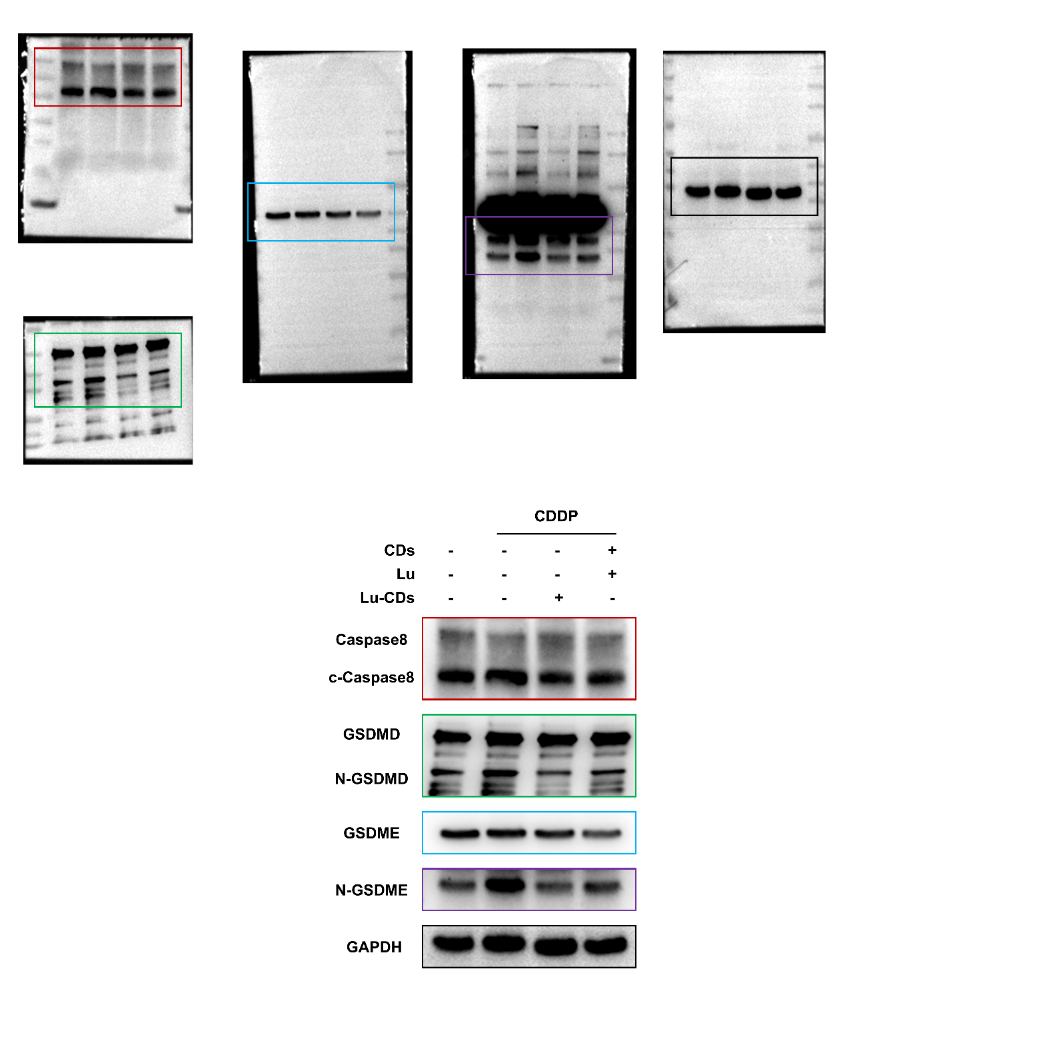


**Figure S30.** Related to **Figure S26**. The uncropped western blot images of the western blot analysis of the expression of PANoptosis biomarkers in HK-2 cells in various experimental groups. The immunoblot bands in the colored box of the uncropped western blot images are the raw data of the processed western blot data marked by the box of the same color.


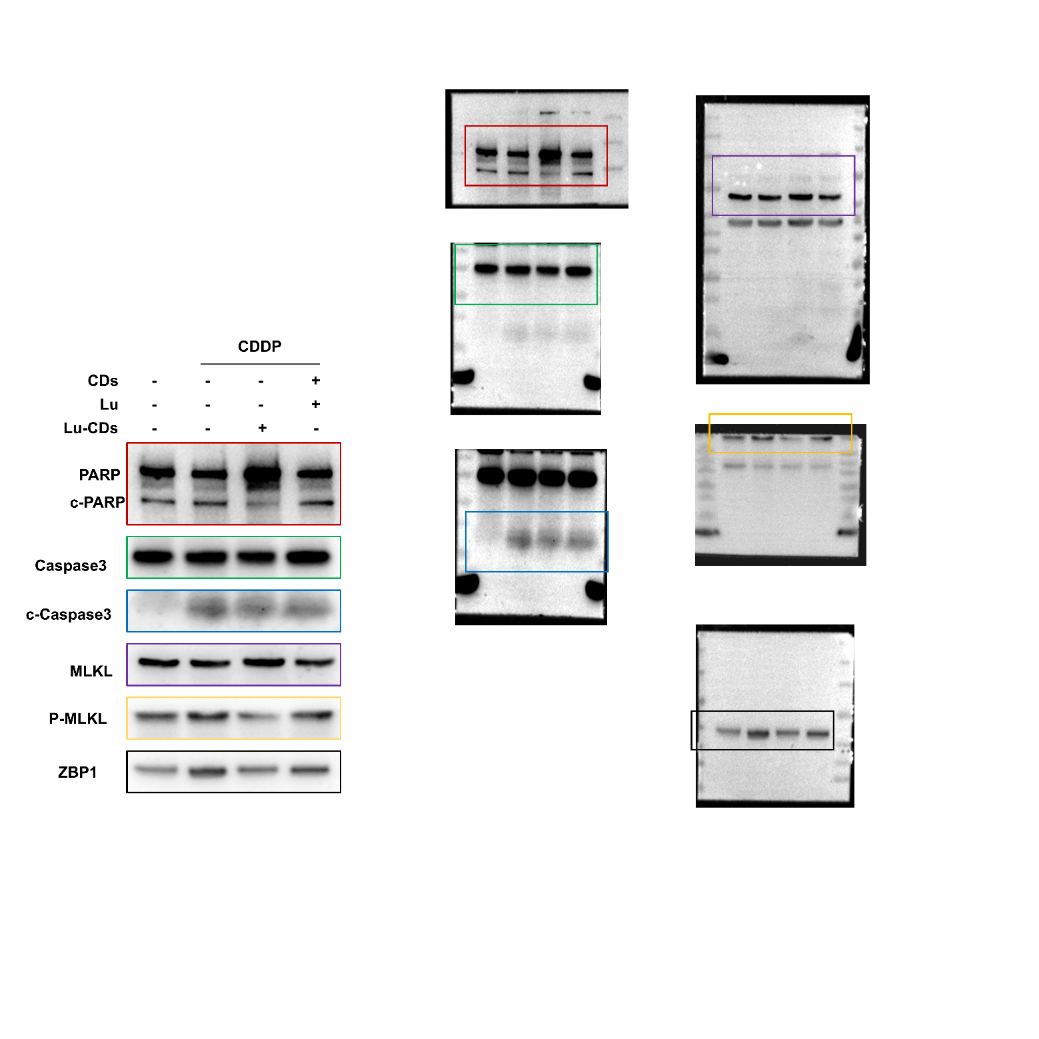


**Figure S31.** Related to **Figure S26**. The uncropped western blot images of the western blot analysis of the expression of PANoptosis biomarkers in HK-2 cells in various experimental groups. The immunoblot bands in the colored box of the uncropped western blot images are the raw data of the processed western blot data marked by the box of the same color.

**III. Tables**

| **Resource type** | **Name** | **Supplier** | **Catalogue Number** | **RRID** |
| --- | --- | --- | --- | --- |
| Antibody | PARP Rabbit Monoclonal Antibody | Beyotime Biotechnology | AF1657 | AB_2920716 |
| Antibody | Caspase 8/P43/P18 Monoclonal Antibody | Proteintech Group, Inc. | 66093-1-Ig | AB_11232214 |
| Antibody | DFNA5/GSDME Monoclonal Antibody | Proteintech Group, Inc. | 67731-1-Ig | AB_2882917 |
| Antibody | GSDMD Monoclonal Antibody | Proteintech Group, Inc. | 66387-1-Ig | AB_2881763 |
| Antibody | ZBP1 Polyclonal Antibody | Proteintech Group, Inc. | 13285-1-AP | AB_3671927 |
| Antibody | Caspase 3 Rabbit mAb | ZEN-BIOSCIENCE | R23315 | AB_3711546 |
| Antibody | [KD]MLKL Rabbit mAb | ZEN-BIOSCIENCE | R380559 | No RRID |
| Antibody | Phospho-MLKL (Ser358) Rabbit mAb | ZEN-BIOSCIENCE | R382136 | No RRID |
| Antibody | TIM1 (KIM-1) Rabbit pAb | ZEN-BIOSCIENCE | 160086 | No RRID |
| Antibody | PPAR alpha Rabbit pAb | ZEN-BIOSCIENCE | 340843 | No RRID |
| Antibody | GAPDH Monoclonal antibody | Proteintech Group, Inc. | 60004-1-Ig | AB_2107436 |
| Antibody | Beta Actin Monoclonal antibody | Proteintech Group, Inc. | 66009-1-Ig | AB_2687938 |

**Table S1.** The detailed catalogue numbers and Research Resource Identifiers (RRIDs) for the antibodies used in the present study.

| **Resource type** | **Name** | **Supplier** | **Catalogue Number** |
| --- | --- | --- | --- |
| Drug | cis-diammineplatinum dichloride (cisplatin, 99.5%) | Aladdin Reagent | C295225 |
| Drug | Luteolin (≥ 98%) | Aladdin Reagent | L107329 |
| Reagent | Citric acid (≥ 99.5%) | Aladdin Reagent | C108869 |
| Reagent | DSPE-mPEG (Mw = 5000) | ToYong Biotechnology | P001008-5K |
| Reagent | DSPE-PEG-Cy5 (Mw = 5000) | ToYong Biotechnology | P006023-5K |
| Reagent | Thiazolyl Blue (MTT) | MedChemExpress | HY-15924 |
| Reagent | Penicillin/streptomycin | Hyclone | SV30010 |
| Reagent | Necrostatin-1 | TargetMol | T1847 |
| Reagent | MCC950 | TargetMol | T3701 |
| Reagent | Z-IETD-FMK | TargetMol | T7019 |

**Table S2.** The detailed catalogue numbers for the drugs and reagents used in the present study.

| **Resource type** | **Name** | **Supplier** | **Catalogue Number** |
| --- | --- | --- | --- |
| Test kit | TUNEL Apoptosis Assay Kit | Beyotime Biotechnology | C1086 |
| Test kit | LDH Cytotoxicity Assay Kit | Beyotime Biotechnology | C0016 |
| Test kit | Beyo3D Calcein/PI Cell Viability/Cytotoxicity Assay Kit | Beyotime Biotechnology | C1371S |
| Test kit | Annexin V-FITC Apoptosis Detection Kit | BestBio | BB-4101 |
| Test kit | Bradford Protein Quantitation Assay Kit | Servicebio | G2001-250ML |
| Test kit | UltraSensitiveTM SP IHC Detection Kit | MXB Biotechnologies | KIT-9720 |
| Test kit | DCFH-DA Kit | Sigma-Aldrich | D6883 |
| Test kit | Super ECL Detection Reagent | Yeasen Biotechnology | 36208ES76 |

**Table S3.** The detailed catalogue numbers for the experimental kits used in the present study.

| **Resource type** | **Name** | **Supplier** | **RRID** |
| --- | --- | --- | --- |
| Cell line | HK-2 (human kidney-2) | ATCC | CVCL_0302 |
| Cell line | LX-2 (human hepatic stellate) | ATCC | CVCL_5792 |

**Table S4.** The detailed Research Resource Identifiers (RRIDs) for the cell lines used in the present study.

**IV. References**

[1] J. P. Perdew, K. Burke, M. Ernzerhof, *Phys. Rev. Lett.* **1996**, *77*, 3865-3868.

[2] S. Grimme, S. Ehrlich, L. Goerigk, *J. Comput. Chem.* **2011**, *32*, 1456-1465.

[3] A. Stukowski, *Model. Simul. Mater. Sc.* **2010**, *18*, 015012.

[4] J. Zeng, D. Zhang, D. Lu, P. Mo, Z. Li, Y. Chen, M. Rynik, L. a. Huang, Z. Li, S. Shi, Y. Wang, H. Ye, P. Tuo, J. Yang, Y. Ding, Y. Li, D. Tisi, Q. Zeng, H. Bao, Y. Xia, J. Huang, K. Muraoka, Y. Wang, J. Chang, F. Yuan, S. L. Bore, C. Cai, Y. Lin, B. Wang, J. Xu, J.-X. Zhu, C. Luo, Y. Zhang, R. E. A. Goodall, W. Liang, A. K. Singh, S. Yao, J. Zhang, R. Wentzcovitch, J. Han, J. Liu, W. Jia, D. M. York, W. E, R. Car, L. Zhang, H. Wang, *J. Chem. Phys.* **2023**, *159*, 054801.

[5] A. P. Thompson, H. M. Aktulga, R. Berger, D. S. Bolintineanu, W. M. Brown, P. S. Crozier, P. J. in 't Veld, A. Kohlmeyer, S. G. Moore, T. D. Nguyen, R. Shan, M. J. Stevens, J. Tranchida, C. Trott, S. J. Plimpton, *Comput. Phys. Commun.* **2022**, *271*, 108171.

[6] F. M. Mourits, F. H. A. Rummens, *Can. J. Chem.* **2011**, *01*, 3007-3020.

[7] A. Laio, M. Parrinello, *Proc. Natl. Acad. Sci. U. S. A.* **2002**, *99*, 12562-12566.

[8] T. D. Kühne, M. Iannuzzi, M. Del Ben, V. V. Rybkin, P. Seewald, F. Stein, T. Laino, R. Z. Khaliullin, O. Schütt, F. Schiffmann, D. Golze, J. Wilhelm, S. Chulkov, M. H. Bani-Hashemian, V. Weber, U. Borštnik, M. Taillefumier, A. S. Jakobovits, A. Lazzaro, H. Pabst, T. Müller, R. Schade, M. Guidon, S. Andermatt, N. Holmberg, G. K. Schenter, A. Hehn, A. Bussy, F. Belleflamme, G. Tabacchi, A. Glöß, M. Lass, I. Bethune, C. J. Mundy, C. Plessl, M. Watkins, J. VandeVondele, M. Krack, J. Hutter, *J. Chem. Phys.* **2020**, *152*, 194103.

[9] S. Grimme, C. Bannwarth, P. Shushkov, *J. Chem. Theory Comput.* **2017**, *13*, 1989-2009.

[10] F. Pietrucci, W. Andreoni, *Phys. Rev. Lett.* **2011**, *107*, 085504.

[11] M. J. Frisch, G. W. Trucks, H. B. Schlegel, G. E. Scuseria, M. A. Robb, J. R. Cheeseman, G. Scalmani, V. Barone, G. A. Petersson, H. Nakatsuji, X. Li, M. Caricato, A. V. Marenich, J. Bloino, B. G. Janesko, R. Gomperts, B. Mennucci, H. P. Hratchian, J. V. Ortiz, A. F. Izmaylov, J. L. Sonnenberg, D. Williams-Young, F. Ding, F. Lipparini, F. Egidi, J. Goings, B. Peng, A. Petrone, T. Henderson, D. Ranasinghe, V. G. Zakrzewski, J. Gao, N. Rega, G. Zheng, W. Liang, M. Hada, M. Ehara, K. Toyota, R. Fukuda, J. Hasegawa, M. Ishida, T. Nakajima, Y. Honda, O. Kitao, H. Nakai, T. Vreven, K. Throssell, J. A. Montgomery, Jr., J. E. Peralta, F. Ogliaro, M. J. Bearpark, J. J. Heyd, E. N. Brothers, K. N. Kudin, V. N. Staroverov, T. A. Keith, R. Kobayashi, J. Normand, K. Raghavachari, A. P. Rendell, J. C. Burant, S. S. Iyengar, J. Tomasi, M. Cossi, J. M. Millam, M. Klene, C. Adamo, R. Cammi, J. W. Ochterski, R. L. Martin, K. Morokuma, O. Farkas, J. B. Foresman, and D. J. Fox, *Gaussian 16 Rev.*, *C.01* Gaussian, Inc., Wallingford CT, **2019**.

[12] K. Wolinski, J. F. Hinton, P. Pulay, *J. Am. Chem. Soc.* **1990**, *112*, 8251-8260.

[13] D. B. Chesnut, K. D. Moore, *J. Comput. Chem.* **1989**, *10*, 648-659.

[14] A. V. Marenich, C. J. Cramer, D. G. Truhlar, *J. Phys. Chem. B* **2009**, *113*, 6378-6396.

[15] Z. Zhu, X. Liu, P. Li, H. Wang, Y. Zhang, M. Liu, J. Ren, *ACS Appl. Mater. Interfaces* **2023**, 15, 21854-21865.

[16] F. Eshraghi-Jazi, M. Nematbakhsh, *J. Toxicol.* **2022**, *2022*, 3507721.
